# The Chromosomal Passenger Protein Birc5b Organizes Microfilaments and Germ Plasm in the Zebrafish Embryo

**DOI:** 10.1371/journal.pgen.1003448

**Published:** 2013-04-18

**Authors:** Sreelaja Nair, Florence Marlow, Elliott Abrams, Lee Kapp, Mary C. Mullins, Francisco Pelegri

**Affiliations:** 1 Laboratory of Genetics, University of Wisconsin–Madison, Madison, Wisconsin, United States of America; 2 Department of Cell and Developmental Biology, Perelman School of Medicine, University of Pennsylvania, Philadelphia, Pennsylvania, United States of America; Princeton University, United States of America

## Abstract

Microtubule-microfilament interactions are important for cytokinesis and subcellular localization of proteins and mRNAs. In the early zebrafish embryo, astral microtubule-microfilament interactions also facilitate a stereotypic segregation pattern of germ plasm ribonucleoparticles (GP RNPs), which is critical for their eventual selective inheritance by germ cells. The precise mechanisms and molecular mediators for both cytoskeletal interactions and GP RNPs segregation are the focus of intense research. Here, we report the molecular identification of a zebrafish maternal-effect mutation *motley* as Birc5b, a homolog of the mammalian Chromosomal Passenger Complex (CPC) component Survivin. The meiosis and mitosis defects in *motley/birc5b* mutant embryos are consistent with failed CPC function, and additional defects in astral microtubule remodeling contribute to failures in the initiation of cytokinesis furrow ingression. Unexpectedly, the *motley/birc5b* mutation also disrupts cortical microfilaments and GP RNP aggregation during early cell divisions. Birc5b localizes to the tips of astral microtubules along with polymerizing cortical F-actin and the GP RNPs. Mutant Birc5b co-localizes with cortical F-actin and GP RNPs, but fails to associate with astral microtubule tips, leading to disorganized microfilaments and GP RNP aggregation defects. Thus, maternal Birc5b localizes to astral microtubule tips and associates with cortical F-actin and GP RNPs, potentially linking the two cytoskeletons to mediate microtubule-microfilament reorganization and GP RNP aggregation during early embryonic cell cycles in zebrafish. In addition to the known mitotic function of CPC components, our analyses reveal a non-canonical role for an evolutionarily conserved CPC protein in microfilament reorganization and germ plasm aggregation.

## Author Summary

We address mechanisms by which germ cell precursors, a cell type that generates sperm and eggs for future generations, are specified in the zebrafish. Germ cell-specific genes are highly conserved across species, and in many animals germ cells are specified by the inheritance of germ plasm, a specialized cytoplasm containing specific proteins and RNAs corresponding to such conserved genes. Germ plasm is inherited as ribonucleoparticles, which are often present in the egg as singletons and which aggregate to generate larger masses that, when inherited by germ cell precursors, will initiate a germ cell-specific gene expression program. Here, we present the functional and molecular analysis of the zebrafish maternal gene, *motley*, which we show encodes a homologue of the Chromosomal Passenger Complex protein Survivin, or Birc5b. We found that, in addition to the expected role of this protein in cell division, characteristic of factors in this complex, Birc5b mediates germ plasm aggregation in the early zebrafish embryo through the coordination of dynamic changes in the cytoskeleton. Our studies provide a mechanistic basis to explain how germ cell determinants are transmitted from one generation to the next and reveal a non-conventional role for a Chromosomal Passenger Complex factor in this process.

## Introduction

A fundamental feature of cell biology is cytoskeletal cross-talk between microtubule and microfilament networks. One key cellular process dependent on these interactions is the positioning of the contractile ring during cytokinesis. Two major groups of microtubules are involved in contractile ring positioning: the center of the mitotic spindle, which resolves into the antiparallel central spindle microtubules, and the poles of the mitotic spindle, which generates astral microtubules. Several studies indicate that central spindle and astral microtubules redundantly stimulate furrowing at the equatorial cortex [1]. Both sets of microtubules must ultimately communicate with cortical microfilaments that form the contractile ring, and the precise mechanism of this communication is an area of intense research. Candidate mediators include the Chromosomal Passenger Complex (CPC), which localizes with chromosomes during metaphase and transitions cortically to the prospective site of membrane ingression during telophase [2], [3]. Loss of CPC function affect two distinct yet related cellular events: chromosomes tend to lag during metaphase, resulting in chromosome segregation errors, and cleavage furrows fail to maintain ingression resulting in cytokinesis failures [4], [5], [6], [7], [8]. Lagging chromosomes can secondarily cause cytokinesis failures during telophase, but analysis of point mutations in CPC proteins reveal independent roles for components of this complex in the initiation of cytokinesis as well [9], [10], [11], in agreement with the localization of the CPC to the early equatorial cortex.

In addition to cytokinesis, a second major requirement of microtubule-microfilament cross-talk is for subcellular localization of proteins and/or mRNAs to either initiate developmental asymmetry during embryogenesis or to achieve a physiological output such as cell migration and axonogenesis. In animal eggs and early embryos, many ribonucleoparticles (RNPs) encode key cell-fate determinants, underscoring the importance of cytoskeletal function in pattern formation during embryogenesis. One such key molecular factor is the germ plasm, a specialized cytoplasm composed of a unique cohort of mRNAs and proteins. In several species including *Drosophila*, *Xenopus*, *C. elegans*, and zebrafish, the primordial germ cells (PGCs) form by selectively inheriting maternally derived germ plasm RNP (GP RNP) complexes [12]. Localization of GP RNPs is best characterized in *Drosophila* where they are transported on microtubules and anchored by microfilaments in a multi-step process that ensures localized germ cell specification [13], [14], [15]. Less is known about GP RNP localization in vertebrate species. Studies in zebrafish suggest that GP RNPs associate with cortical microfilaments, which organize in a microtubule-dependent manner into circumferential concentric rings that facilitate germ plasm aggregation [16]. However, the precise molecular mechanism(s) of cytoskeletal cross-talk that mediates this reorganization remain unknown.

Here, we describe a zebrafish maternal-effect mutant *motley* and identify it as *birc5b*, a zebrafish homolog of the mammalian CPC protein, Birc5/Survivin. Birc5b is subcellularly localized as a CPC protein and *motley/birc5b* mutants display meiotic and mitotic chromosome segregation errors and cell division phenotypes characteristic of failed CPC function. Additionally, *motley/birc5b* mutants fail to initiate cytokinesis furrow ingression as reflected by defects in astral microtubule reorganization at incipient furrows, confirming an early role for a CPC protein in furrow formation. Unexpectedly, *motley/birc5b* mutants also exhibit defects in microfilament reorganization in the embryo prior to initiation of the first cytokinesis furrow, and these defects are accompanied by a failure in GP RNP aggregation. In wild-type embryos, Birc5b protein localizes to the tips of astral microtubules contacting the cortex, where it also co-localizes with actin and GP RNPs. We propose a model in which Birc5b at astral microtubule tips mediates microtubule-microfilament interaction to achieve reorganization of cortical microfilaments and facilitate GP RNP aggregation prior to and during cytokinesis furrow initiation.

## Results

### *motley* is an essential maternal factor required for DNA segregation and cytokinesis during early zebrafish development

**Figure 1:**
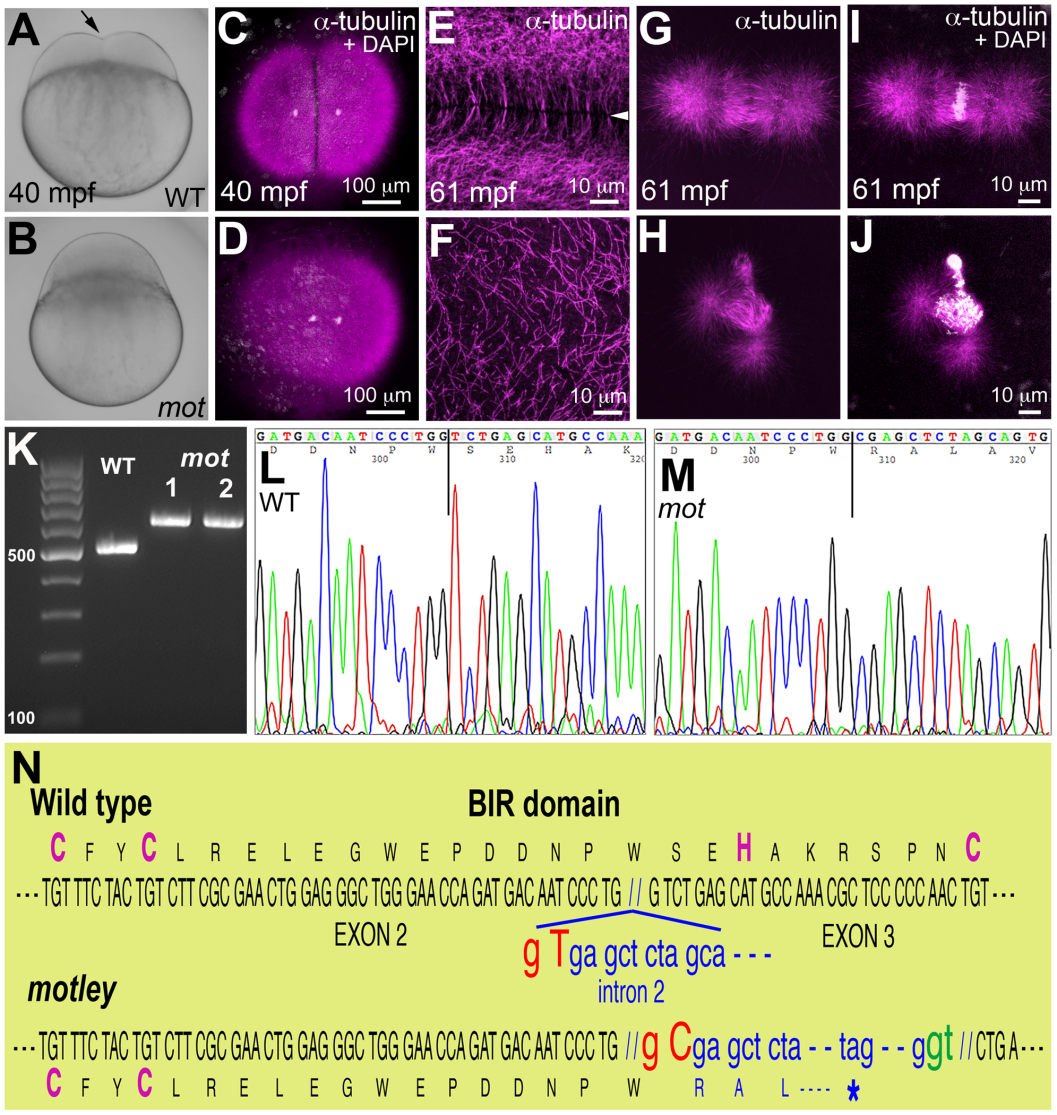
*motley* is a mutation in *birc5b*, which causes early cell division defects in zebrafish embryos. The cleavage furrow (arrow) seen in live wild-type embryos at 40 mpf during the first cell cycle (A) is absent in *motley* mutants (B). Early embryonic cytokinesis in zebrafish are rapid and overlap with each other with furrow completion of the previous cell cycle occurring concurrently with the furrow initiation of the next. This results in furrows of various stages of development being present at a single time point in the same embryo (e.g. Figure S1E). During the early cell cycles, immunolabeling for α-tubulin reveal abutting microtubule arrays between nuclei in wild-type blastodisc (C, shown for the furrow corresponding to the first cell cycle), which are abnormal in *motley* (D). DAPI channel in (C,D) reveals nuclear cycle progresses normally in mutants, although separation of daughter nuclei is reduced likely due to spindle defects (scattered white speckles are caused by autofluorescence by yolk granules, which typically can not be fully removed in the mounting preparation). High magnifications shown here corresponding to the second cell cycles in wild-type and *motley* show the microtubule-free zone at the furrow in wild-type blastodisc (arrowhead in E, low magnification in Figure S1A), which does not exist in *motley* (F, low magnification in Figure S1C, boxed area rotated −90° in F). Wild-type mitotic spindle aligns the DNA at the center of the spindle (G, I) while spindles in *motley* mutants are bent with the DNA spread along their length (H, J). RT-PCRs show that wild-type *birc5b* is ∼500 bp, while *motley* allele from two different homozygous mutant females is larger at ∼600 bp (K). Sequence chromatograms of wild-type (L) and *motley* (M) *birc5b* alleles show that the *motley* sequence is unaltered up to position 236 from the start ATG (vertical line, corresponding to base 305 in the shown sequence chromatograms) and thereafter corresponds to intronic sequence. Schematic of the Birc5b BIR domain that is disrupted due to intron insertion in *motley* (N).

The mutations *motley* (*mot^p1aiue^*) and *p4anua*, isolated in an ENU-induced mutagenesis screen for recessive zebrafish maternal-effect genes [17] display early cytokinesis defects in the embryo (Figure 1A–1F; Figure S1A–S1H). In this study, we present the identification and characterization of the *motley* mutation. Homozygous *motley* females mature into viable, fertile adults. However, embryos from such females (*motley* mutants herein) manifest a completely penetrant cell division defect, which results in lethality at ∼4 hours post fertilization (hpf). Live *motley* mutants were indistinguishable from wild-type embryos during the first 30 minutes post fertilization (mpf). However, shortly after, when the first cytokinesis furrow became visible in wild-type blastodiscs, *motley* mutant blastodiscs lacked a membrane indentation characteristic of furrow formation (Figure 1A, 1B). In early wild-type embryos at telophase, when furrow initiation occurs, immunolabeling for α-tubulin revealed arrays of microtubules at the incipient furrow during the first cell cycle (Figure 1C), which were absent in *motley* though karyokinesis appeared to have progressed (Figure 1D). In wild-type embryos, at this stage in furrow formation, a microtubule-free zone appears between abutting arrays of bundled microtubules at incipient furrows (shown for the second cell cycle in Figure 1E). In *motley* mutants at the same developmental stage, astral microtubules failed to bundle opposite each other resulting in a disorganized mesh (Figure 1F). By 2hpf, the cell adhesion molecule β-catenin accumulated at mature cleavage furrows in wild-type embryos (Figure S1B), a pattern that was absent in *motley* mutants (Figure S1D). Though DNA segregates in *motley* mutants initially (Figure 1D), the mitotic spindle itself was abnormal. In wild-type embryos, bipolar mitotic spindles always aligned sister chromatids at the metaphase plate (Figure 1G, 1I). In *motley* mutants, mitotic spindles were typically bent with chromosomal DNA aberrantly spread along its length (Figure 1H, 1J). DNA segregation defects in *motley* eventually manifested as unevenly distributed nuclear masses and chromosomal bridges by 2hpf (Figure S1D).

### The *motley* lesion is an exon-intron junctional mutation in zebrafish *birc5b*

Linkage analysis mapped the *motley* locus to the zebrafish chromosome 23 where it was fully linked to the Simple Sequence Length Polymorphism (SSLP) marker z14967 in 565 meioses. We tested *birc5b*, a gene present in the vicinity of z14967, as the candidate locus affected in *motley* mutants. *birc5b* transcripts from wild-type eggs were of the expected ∼500 base pair (bp) size, however *birc5b* transcripts from *motley* eggs were ∼100 bp larger, indicating aberrant splicing (Figure 1K). Sequencing both transcripts revealed a single T to C transition at the highly conserved splice donor base pair GT in the second intron of the *motley* allele (Figure 1L–1N). This mutation results in alternative splicing at the GT base pair of a cryptic splice donor site 113 bps into the second intron (Figure 1N). While wild-type Birc5b protein is predicted to be a 144 amino acid product, the *motley* mutant allele is expected to result in a truncated Birc5b protein containing the first 79 wild-type residues followed by mis-translation from the point of intron insertion, yielding a mis-translated, truncated protein of 111 residues. The CX_2_CX_16_HX_6_C signature BIR domain of Birc5b spans the second and third exons and the mutation disrupts the BIR domain from residue S80 onwards, resulting in loss of the C-terminal part of the BIR domain and protein, including conserved H82 and C89 residues within the BIR domain (Figure 1N). Protein sequence analyses indicate that *motley/*Birc5b is a zebrafish homologue of the Baculoviral IAP protein, Birc5/Survivin (Figure S1L). The two paralogues in zebrafish, Birc5a and Birc5b are 51.4% and 46.8% similar to human BIRC5/Survivin, respectively (Figure S1L). Consistent with previous observations that both paralogues were maternally expressed [18] we isolated both *birc5a* and *birc5b* transcripts from wild-type embryos during early development at 1 and 4hpf (Figure S1I). RT-PCR analysis indicates that *birc5a* continues to be expressed during development at 24hpf and beyond, whereas *birc5b* transcripts were undetectable at these later stages (Figure S1I).

### The *motley/birc5b* mutation causes meiosis defects in mature zebrafish eggs

**Figure 2:**
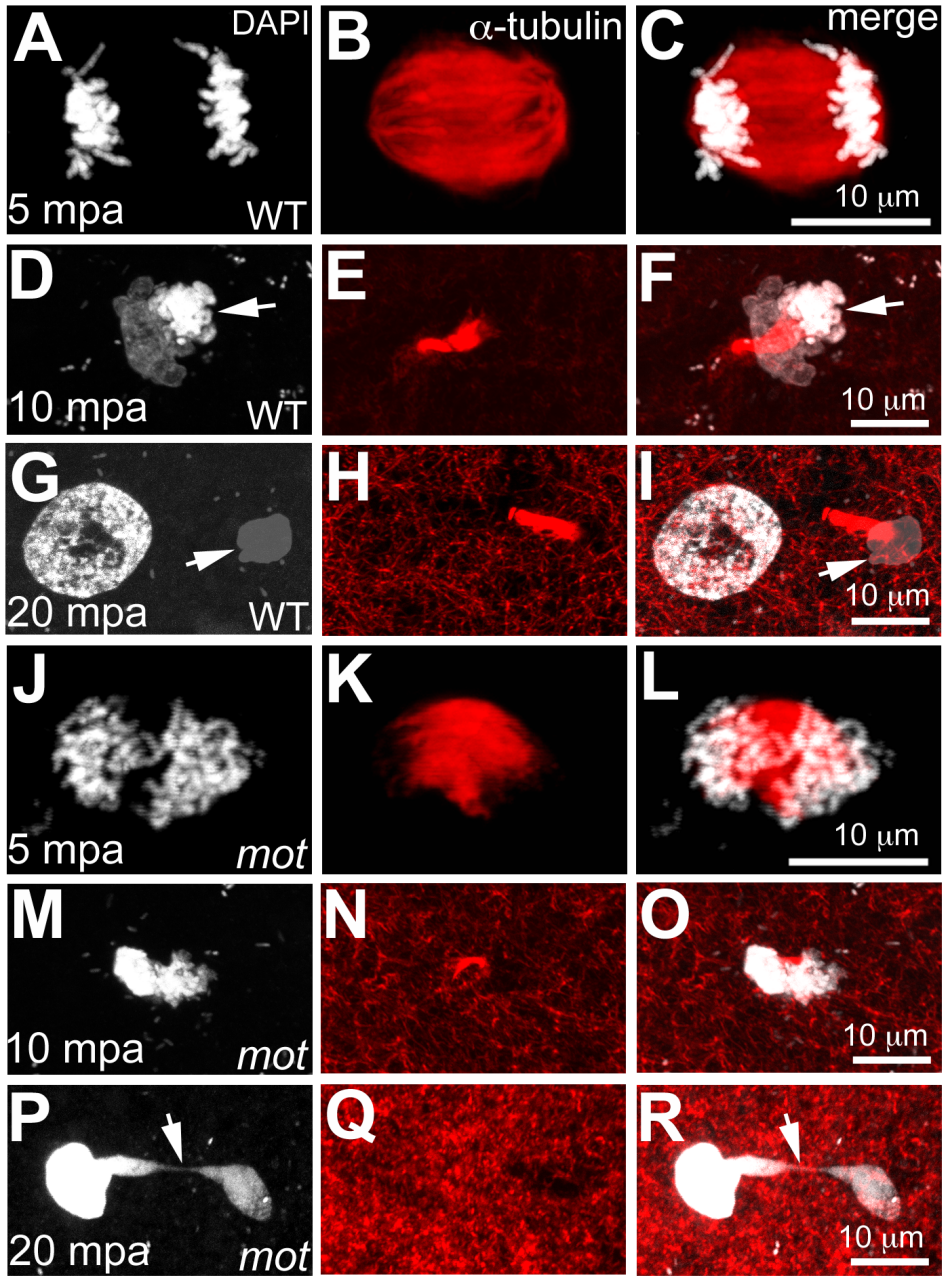
*motley* mutant eggs fail to complete meiosis II. In water-activated wild-type eggs, sister chromatids align at meiotic spindle poles during anaphase (A–C). The meiotic spindle bundles into a nascent midbody between one set of condensing (D, F arrow) and one set of decondensing chromatid sets (D–F). The smaller, condensed polar body (G, I arrow) and the decondensed, larger female pronucleus separate with the meiotic midbody attached to the polar body (G–I). In *motley* eggs, sister chromatids spread along the bent meiotic spindle (J–L). Sister chromatid sets cannot be distinguished as pronucleus or polar body and a smaller meiotic midbody is occasionally seen in *motley* (M–O). Sister chromatids attempt to separate but remain connected by chromosomal bridges spanning several microns (P–R, arrows).

**Figure 3:**
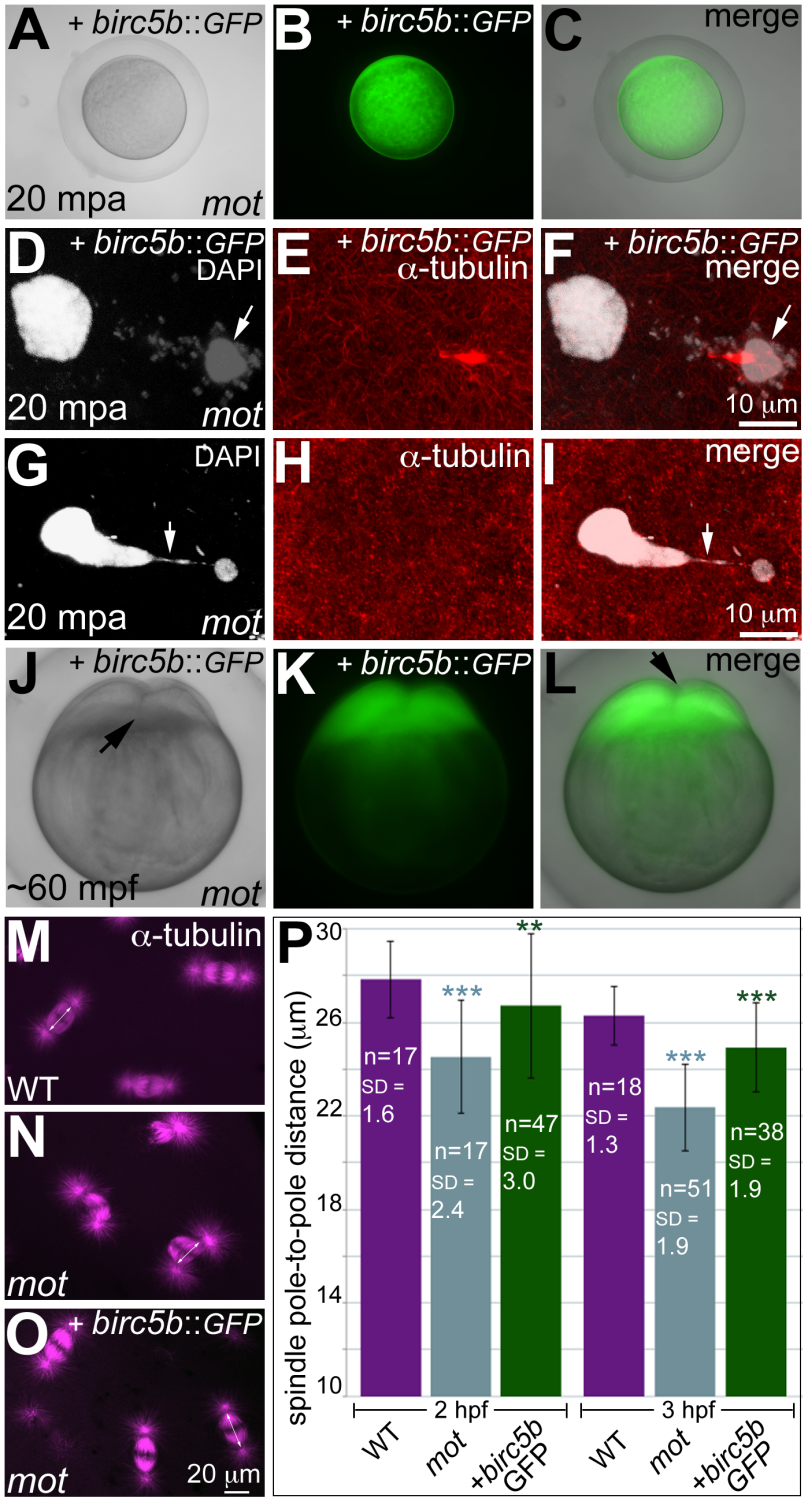
Wild-type *birc5b* rescues cell division defects in *motley* mutants. In vitro cultured oocytes from homozygous *motley* females express the injected *birc5b*::*eGFP*, mature and water-activate normally (A–C). Birc5b::eGFP-expressing *motley* eggs successfully complete meiosis as seen by the presence of meiotic midbody (E, F) and a distinctive polar body (D, F arrow). Control uninjected *motley* eggs do not have a meiotic midbody and form chromosomal bridges (G, I arrow) between condensed DNA masses (G–I). Normal cleavage furrows at ∼60 mpf in in vitro fertilized embryos from Birc5b::eGFP-expressing eggs (J–L, arrows). Normal mitotic spindles in wild-type embryos (M), bent spindles in *motley* mutants (N), and mitotic spindles in Birc5b::eGFP-expressing *motley* embryos resembling wild-type (O). Due to the bending of the spindle, linear spindle pole-to-pole distance (SPD) is shorter in *motley* mutants than wild-type, while in Birc5b::eGFP-expressing *motley* embryos this distance approaches wild-type (P). Double-headed arrows in M-O indicate example SPD distances used for quantitation in P. Error bars in P represent Standard Deviation. Statistical significance of SPD values was ascertained by t-test (2-tailed, unpaired, unequal variance). P<0.001 = ***, P<0.01 = **. SPD distances were compared between uninjected *motley* and wild-type at 2 and 3 hpf (light blue asterisks), and between uninjected *motley* and *birc5b::GFP* injected *motley* at 2 and 3 hpf (green asterisks). P values were not significant (P>0.05), between wild-type and rescued *birc5b::GFP* injected SPDs.

Survivin and its homologs are required for meiosis in both vertebrates and invertebrates [5], [19], [20], which prompted us to assay for meiosis defects in eggs from *motley* mutant females (Figure 2). Mature zebrafish eggs are arrested at metaphase of meiosis II, and egg activation, which occurs upon contact with water, results in meiosis resumption and release of the second polar body. We compared this process in unfertilized water-activated eggs from wild-type and homozygous *motley* females by a time-course analysis of meiosis II completion. In wild-type eggs at 5 minutes post activation (mpa), a meiotic spindle can be observed with the sister chromatids aligned at the spindle poles during anaphase (Figure 2A–2C). By 10mpa, the spindle apparatus bundles as the meiotic midbody between one set of condensing sister chromatids and a second set of decondensing sister chromatids (Figure 2D–2F). By 20mpa, a nuclear membrane forms around the decondensed DNA to generate the female pronucleus, while the condensed DNA remains tightly associated with the meiotic midbody and becomes the polar body (Figure 2G–2I). In *motley* eggs at the same time points, the meiotic spindle exhibits defects similar to those of its mitotic counterpart. At 5mpa, the meiotic spindle in *motley* eggs is bent and chromosomes spread along the spindle instead of being aligned at the poles (Figure 2J–2L). At 10mpa an incipient midbody-like microtubule bundle forms only occasionally between sister chromatid sets, both of which appear equally condensed in contrast to wild-type (Figure 2M–2O). At 20mpa, a meiotic midbody is not observed in *motley* eggs and both resulting nuclei appear highly condensed and connected by chromosomal bridges (Figure 2P–2R; Figure 3G–3I). Thus, in addition to its role in mitosis, maternal Birc5b is also required for meiotic spindle organization and successful meiosis completion. Despite defects in the completion of meiosis II and the aberrant appearance of the female pronucleus, the oocyte nucleus from *motley* mutants is able to fuse with the male pronucleus in fertilized embryos to form the zygotic nucleus (data not shown).

### Exogenous wild-type *birc5b* rescues meiosis, mitosis, and cytokinesis phenotypes in *motley* mutants

We validated that the molecular lesion in *motley* affected Birc5b function by providing *motley* eggs and embryos with exogenous wild-type *birc5b* mRNA (Figure 3; Figure S2). To assay for meiosis rescue in *motley* eggs, immature stage IV oocytes were isolated from homozygous *motley* females and microinjected with wild-type *birc5b::eGFP* mRNA. *birc5b::eGFP* mRNA injected *motley* oocytes express Birc5b::GFP fusion protein within 1 hour post injection (hpi) (Figure S2A–S2C). At 3hpi, *motley* oocytes had fully matured into translucent eggs that continued to exhibit strong Birc5b::GFP protein expression (Figure S2D–S2F), and activated normally upon contact with water (Figure 3A–3C). In these Birc5b::GFP-expressing *motley* eggs, we were able to unambiguously detect a distinct larger female pronucleus and a condensed polar body with its associated meiotic midbody (Figure 3D–3F), indicating that both female pronuclear decondensation and midbody formation defects were rescued. In contrast, *motley* eggs derived from an uninjected subset of oocytes exhibited two highly condensed nuclear bodies connected by chromosomal bridges and had no detectable meiotic midbody (Figure 3G–3I), similar to the phenotype observed in eggs that mature within homozygous *motley* females (Figure 2P–2R).

A subset of *motley* eggs injected with *birc5b::eGFP* mRNA during in vitro oogenesis were in vitro fertilized to assay for rescue of post-fertilization mitotic phenotypes. In such Birc5b::GFP-expressing *motley* embryos, normal cytokinesis furrows corresponding to the first two cell cycles were observed at ∼60mpf (Figure 3J–3L). These results indicate that exogenous wild-type *birc5b::eGFP* injected into immature oocytes rescues *motley* mutant phenotypes both during oogenesis and early embryogenesis.

We also observed late rescue of *motley*-associated phenotypes when *birc5b::eGFP* mRNA was injected into embryos at the 1-cell stage. At ∼2hpf several cytokinesis furrows were observed in *motley* mutants expressing Birc5b::GFP (Figure S2G–S2I), whereas uninjected sibling *motley* embryos did not exhibit cleavage furrows at any stages (Figure S2J). The timing of furrow formation in *birc5b::GFP* injected *motley* embryos, ∼80 minutes after the normal initiation of cell division in wild-type embryos, coincides with the appearance of Birc5b::GFP fluorescence in injected embryos and likely reflects a lag in translation of the injected mRNA to generate sufficient protein for rescue. We characterized this late-stage cell division rescue by comparing the spindle shape, linear spindle pole-to-pole distance (SPD, as a measure of spindle bending) and the presence of midbodies between Birc5b::GFP-expressing *motley*, uninjected siblings and wild-type embryos. At 2hpf, wild-type mitotic spindles were normal and SPD measured ∼27.8 µm (Figure 3M, 3P), while in *motley* embryos the spindles were bent with a significantly reduced SPD of ∼24.5 µm (Figure 3N, 3P). In *motley* mutants expressing Birc5b::GFP, mitotic spindle morphology was rescued to wild-type and the SPD measured ∼26.7 µm approaching the wild-type measurements (Figure 3O, 3P). Additionally, midbody formation was also rescued in *motley* mutants injected with *birc5b::GFP* at the 1-cell stage. During cleavage stages in wild-type embryos, midbodies were readily observed (Figure S2L), while midbodies were never observed in *motley* embryos due to failed cytokinesis (Figure S2M). However, in Birc5b::GFP-expressing *motley* embryos midbodies were detected, indicating that the cytokinesis furrows seen in live *motley* mutants at ∼2hpf could transition into mature cleavage furrows (Figure S2N, S2O). Thus, exogenous Birc5b can rescue all aspects of the mutant phenotypes observed in *motley* embryos during meiosis II and early embryonic cell divisions, confirming that the locus affected in *motley* is *birc5b*.

Since both *birc5a* and *birc5b* are maternally present in zebrafish embryos as mRNA (Figure S1I; [18]) and protein (Figure S3A; see below), we asked whether these duplicate genes might share a common function. In contrast to the case with Birc5b::GFP protein, expression of Birc5a::GFP during oogenesis is unable to rescue the *motley/birc5b* mutant phenotype (Figure S3B–S3J). This is in agreement with the observed fully-penetrant maternal-effect phenotype caused by the *motley/birc5b* mutation in spite of the fact that Birc5a expression is not affected in this background (Figure S1 and data not shown). Together, these data suggests subfunctionalization for the Birc5a and Birc5b paralogs with respect to cytokinesis in the early embryo.

### Birc5b protein localizes to the meiotic and mitotic spindles and to the site of membrane ingression during cytokinesis

Prior to ascertaining the subcellular localization of Birc5b protein by immunolabeling, we identified antibodies that specifically recognized this protein. We performed western blot analysis using two commercially available monoclonal antibodies: anti-Survivin, developed against full-length human Survivin, and anti-Survivin-BIR, developed against the BIR domain present in human Survivin. Our data indicate that the anti-Survivin and anti-Survivin-BIR antibodies recognize Birc5a and Birc5b, respectively, without detectable non-specific cross-reactivity (Figure 3SA).

**Figure 4:**
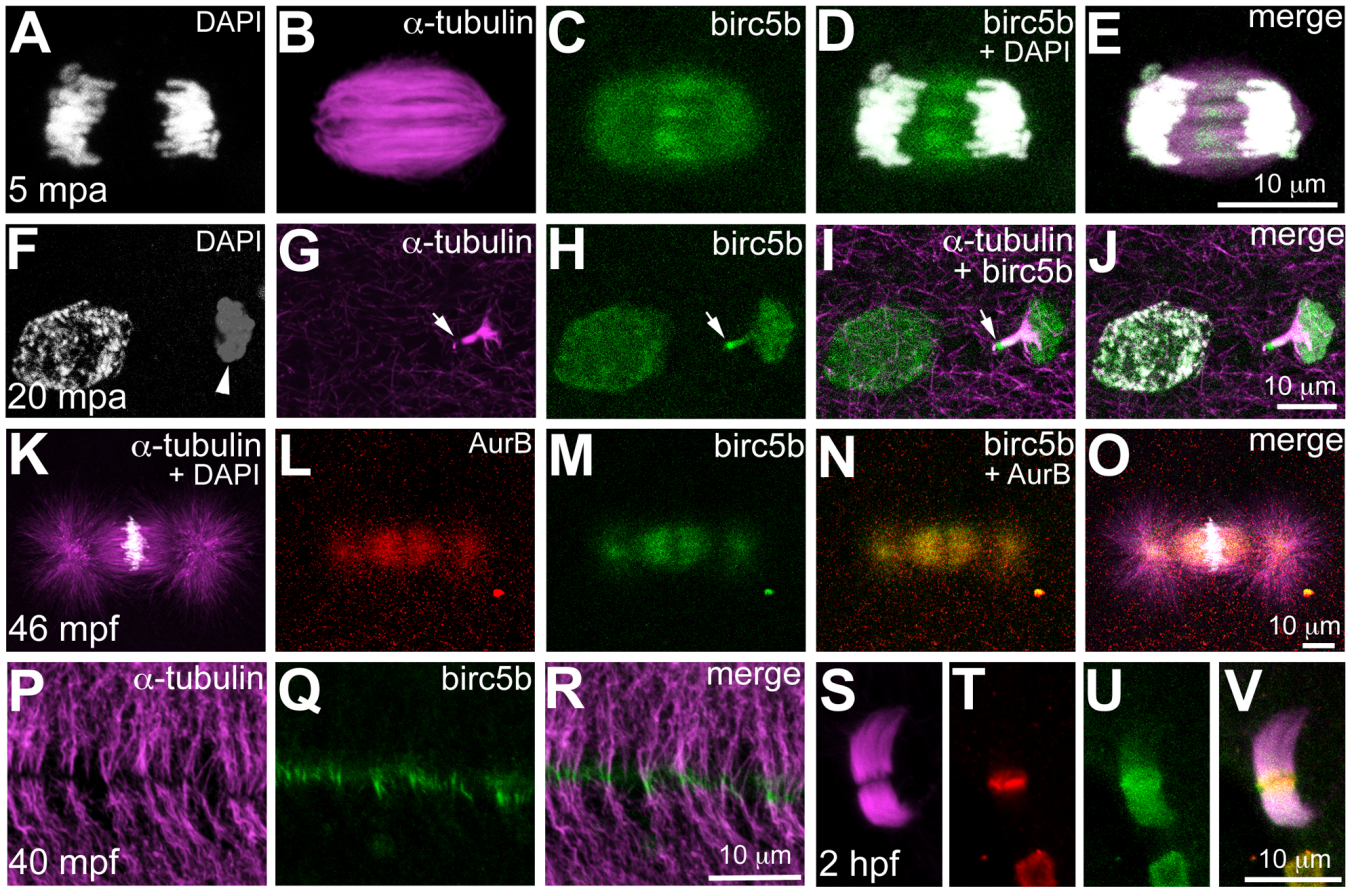
Birc5b protein is expressed during meiosis, mitosis, and cytokinesis. Birc5b is expressed along the length of the meiotic spindle during anaphase and concentrates at the spindle midzone (A–E). Birc5b localizes to the meiotic midbody and concentrates at one end, corresponding to the ooplasmic side (F–J, arrow in H, I). During mitotic metaphase Birc5b co-localizes with AurB on the spindle (K–O). During cytokinesis in the early embryo, Birc5b localizes to the tips of bundled microtubules at the furrow (P–R). At the midbody in the cleavage-stage embryo, shown here at 2 hpf (S–V), Birc5b expression (U) overlaps with AurB (T) at the center of bundled microtubules (S) and extends beyond it.

We assayed the subcellular localization of Birc5b using the anti-Survivin-BIR antibody, which our western analysis suggests is specific to Birc5b. Immunolabeling during meiotic anaphase in wild-type eggs at 5mpa revealed that Birc5b protein localized to the central region of the meiotic spindle (Figure 4A–4E). At 20mpa, the meiotic midbody associates with the forming polar body (Figure 4F–4J). Interestingly, Birc5b protein always localized distinctly to the end of the meiotic midbody farthest from the polar body, presumably at the site of meiotic cytokinesis (Figure 4H–4J). During early mitosis in the embryo, co-immunolabeling with the CPC protein Aurora Kinase B (AurB) revealed that Birc5b co-localized with AurB at the mitotic spindle during metaphase (Figure 4K–4O). During cytokinesis, Birc5b translocated to the cortex, where it localized to bundled microtubule ends abutting at the incipient cleavage furrow (Figure 4P–4R). During these early cell divisions, AurB also localizes to the bundled tips of microtubules at the furrow [21], along with Birc5b (data not shown). Both Birc5b and AurB continued to colocalize in midbodies during mid-cleavage stages at 2hpf (Figure 4S–4V).

The subcellular expression of Birc5b protein is consistent with its inferred function as a CPC protein required for DNA segregation, spindle morphology, and cytokinesis during meiosis and mitosis in the early zebrafish embryo. Additionally, localization of Birc5b to the tips of bundled microtubules is consistent with an additional role for this CPC protein in facilitating microtubule remodeling for initiating furrow ingression, in agreement with previous studies [9], [10], [11].

### The *motley/birc5b* mutation affects microfilament reorganization prior to the first embryonic mitosis

**Figure 5:**
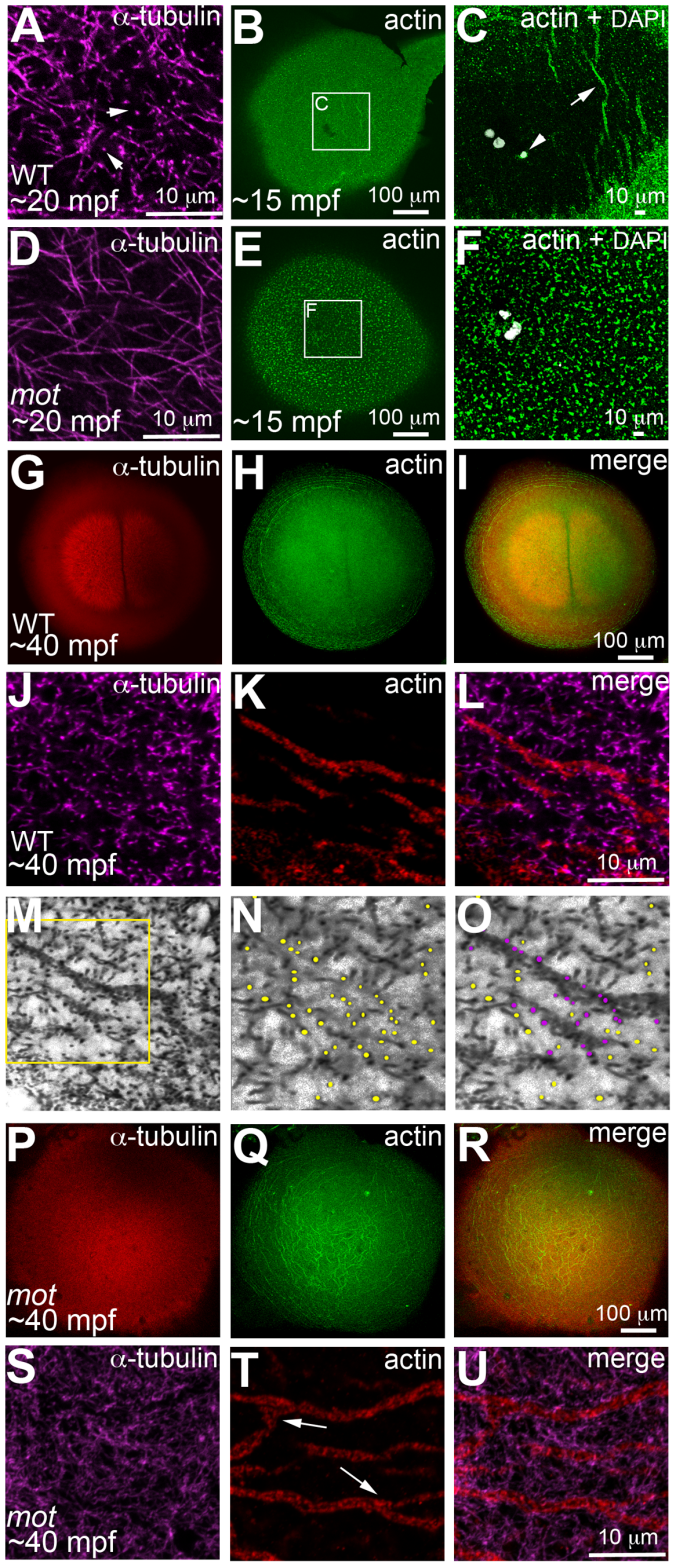
*motley/birc5b* mutants fail to rearrange cortical actin microfilaments. Animal views of blastodisc cortex. In wild-type embryos (A–C) tips of the sperm monoaster microtubules are found at the cortex (A, arrows). Microtubule punctae are seen at the cortex, which are the ends of astral microtubules present in focal planes beneath the cortex (not shown). Short F-actin seed filaments polymerize (C, arrow) and clear from the center of the wild-type blastodisc (B, C; polar body, arrowhead in C), and congressing pronuclei are also apparent). In *motley/birc5b* mutants, sperm monoaster microtubules tips cannot be detected at the cortex (D), and polymerizing F-actin seed filaments fail to clear from the center (E, F). At 40 mpf in wild-type embryos, astral microtubules of the first mitotic spindle radiate towards the blastodisc cortical periphery (G) while the microfilaments are seen at the cortical periphery in concentric rings (H, I). The tips of the mitotic aster microtubules are also seen at the cortex at this time, (J) where they contact peripheral microfilaments (K, L). Panel M is a color-inverted black and white image of J, a region of which has been expanded in (N,O). In (N), microtubule tips in J and M are indicated by yellow dots, while in (O) microtubule tips from N that additionally appear in contact with the microfilaments have been colored magenta. Mitotic astral microtubules fail to separate in *motley* (P) and the microfilaments are found aberrantly in the center of the cortex and are branched (Q, R). The tips of *motley* mitotic aster microtubules are also absent at the cortex (S), while microfilaments in *motley* are aberrantly branched (T, U, arrows in T indicate ectopic branching) and do not appear to colocalize with microtubules (U). Panels C and F are high magnifications of indicated boxes in B, E, respectively. Panels A, D, (J–O) and (S–T) are high magnifications of the cortex from blastodiscs of stages comparable to those in B, E, I and R, respectively. Reduced labeling intensity of the right aster in G is an imaging artefact due to slight tilting of the blastodisc within the semi-flat mount preparation.

In zebrafish embryos, prior to and during the first mitosis, the microfilament and microtubule cytoskeletons grow dynamically in a concerted manner, which is evident in the reorganization of cortical microfilaments before the end of the first zygotic cell cycle ([16]; Figure 5). Immediately upon fertilization, the sperm derived centrioles nucleate a sperm monoaster near the fusing pronuclei, microtubules from which grow towards the cortex [16]. 0.5 µm optical cross-sections through the wild-type blastodisc cortex reveal monoastral microtubule tips suggesting that the majority of microtubules grow and terminate at the cortex (Figure 5A). During the same time-frame, F-actin seed filaments at the center of the blastodisc cortex move towards the periphery creating an actin-free zone (Figure 5B, 5C). In *motley/birc5b* mutants, a 0.5 µm section through the blastodisc cortex reveals sperm monoaster microtubules, which are aberrantly located along the cortical plane, and an absence of microtubule tips at the cortex (Figure 5D). Analysis of the actin cytoskeleton during this time-frame show that F-actin seed filaments fail to clear from the center of the blastodisc cortex; instead the cortex remains mottled with F-actin seed filaments (Figure 5E, 5F). As the sperm monoaster disappears, the first embryonic mitosis proceeds in wild-type embryos and the mitotic spindle poles resolve into sets of astral microtubules, which like microtubules of the sperm monoaster, also radiate towards the blastodisc cortex (Figure 5G). Again, coincident with the cortical astral microtubule growth, cortical F-actin organizes into concentric rings at the blastodisc periphery (Figure 5H, 5I; [16]). Higher magnification views of the two cytoskeletons reveal microtubule tips at the cortex in a 0.5 µm optical section (Figure 5J, 5M, 5N), a subset of which are in contact with cortical microfilaments arranged in unbranched concentric rings at the periphery (Figure 5K, 5L, 5O). During the first zygotic mitosis in *motley/birc5b* mutant embryos, spindle pole astral microtubules reach the cortex, but as described earlier (Figure 1D), fail to resolve into the two sets (Figure 5P) characteristic of the first mitosis (Figure 1C, Figure 5G). Furthermore, similar to the sperm monoaster microtubules, in *motley/birc5b* mutants, spindle pole astral microtubule tips were also not detectable at the cortex (Figure 5S). Analysis of the cortical microfilaments during the first zygotic mitosis in *motley/birc5b* reveal that the microfilaments fail to organize into peripheral rings and are instead found ectopically in the center of the blastodisc cortex (Figure 5Q, 5R). Higher magnifications of the cortical cytoskeleton additionally revealed that the microfilaments are branched and as the microtubule ends are not seen at the cortex, do not colocalize with the tips (Figure 5T, 5U). These cortical cytoskeletal defects indicate a role for maternal Birc5b in microtubule dynamics at the blastodisc cortex, and an additional novel role for this CPC protein in cortical actin cytoskeleton rearrangements prior to the first zygotic mitosis.

### The *motley/birc5b* mutation causes a failure in germ plasm RNP multimerization

Because of the postulated role for the cortical cytoskeleton on zebrafish germ plasm localization [16] and the function of *motley/birc5b* in cortical cytoskeletal reorganization, we tested whether germ plasm RNP segregation is affected in *motley/birc5b* mutants. We first corroborated that during the early embryonic cell divisions, germ plasm mRNAs such as *nanos* localize to cortical microfilaments at the blastodisc periphery (Figure S4), as had been previously posited [16]. We also discovered that an antibody against the human phosphorylated non-muscle myosin (NMII-p) labeled the distal furrow where GP RNPs are recruited in the 2- (Figure S5A–S5C) and 4-cell embryos. Double labeling experiments showed that the anti-NMII-p label co-localized with germ plasm mRNAs *vasa*, *dead end* (*dnd*) and *nanos* at the furrow as well as to non-furrow regions at the cortical periphery (Figure S5D–S5U). The functional relevance of the anti-NMII-p label to GP RNPs is currently under investigation, however, these observations indicated that the anti-NMII-p antibody serves as a convenient probe to detect GP RNPs in the early zebrafish embryo. We infer from the localization of NMII-p with all three tested germ plasm mRNAs that most GP RNPs may contain the same basic molecular components.

In wild-type embryos immediately upon fertilization (Figure S6A; [16]) and in unfertilized embryos (data not shown), GP RNPs are distributed as a broad cortical band surrounding a GP RNP-free zone at the center of the blastodisc. The underlying basis for this initial distribution is not known but may reflect intrinsic differences in the egg cortex established during oogenesis. Upon fertilization, the GP RNP-free zone is seen to expand outwardly, through a process we have previously proposed involves microtubules from the sperm monoaster and the spindle pole asters pushing growing microfilaments and associated GP RNPs away from the center of the blastodisc, generating an increasingly narrow and more peripherally located band of GP RNPs and microfilaments (Figure S6B, S6C; [16]). A failure in this proposed process is reflected in *motley/birc5b* mutants (Figure S6) and nocodazole-treated embryos (data not shown), where the initial broad cortical band of GP RNPs remain unaffected (Figure S6D), but the central GP RNP-free zone does not appear to expand, so that aggregates continue to exhibit a broad cortical distribution (Figure S6E, S6F) similar to that observed in the egg/embryo immediately after activation/fertilization. As expected from the furrow initiation defect, GP RNPs do not undergo furrow recruitment in *motley/birc5b* mutants (Figure S6E, S6F). In situ hybridization analysis to detect GP RNAs indicates similar defects in *motley* mutants (Figure S6J, S6K and data not shown). Together, these observations indicate that *motley/birc5b* mutants have defects in GP RNP segregation prior to and independent of their recruitment to the cleavage furrows.

**Figure 6:**
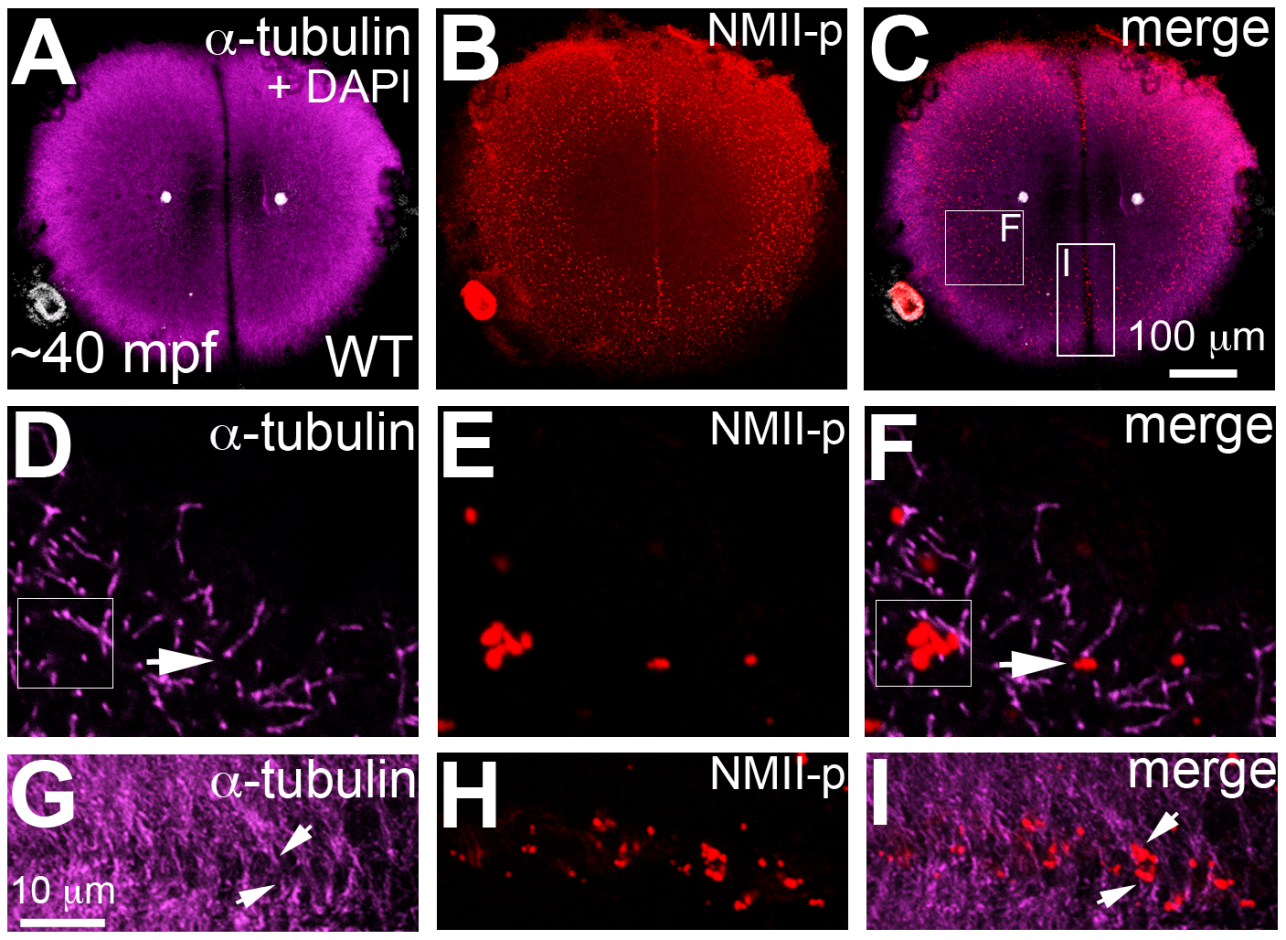
Germ plasm RNPs localize to the tips of astral microtubules. Animal views of blastodisc cortex. (A–C) GP RNPs are located in a band (B) at the cortical periphery. Higher magnification images indicate that multimerized GP RNPs are located at the tips of spindle pole astral microtubules (D–F; G–I). In non-furrow peripheral regions (D–F), several microtubule tips (D) can be observed to coincide with a large multimerized GP RNP (E, F). Arrow in F points to a smaller multimerized aggregate at a microtubule tip. At the cleavage furrow (G–I), multimerized GP RNPs are found as bilateral rows of aggregates (arrows in I) at bundled tips of astral microtubules from contralateral sides (G–I). Panels G–I are high magnification views of the rectangular box (labeled I) in panel C, rotated 90° counterclockwise. Panels D–F are high magnification views from an embryo comparable to that in C of a non-furrow region similar to the square box indicated in panel C (labeled F).

**Figure 7:**
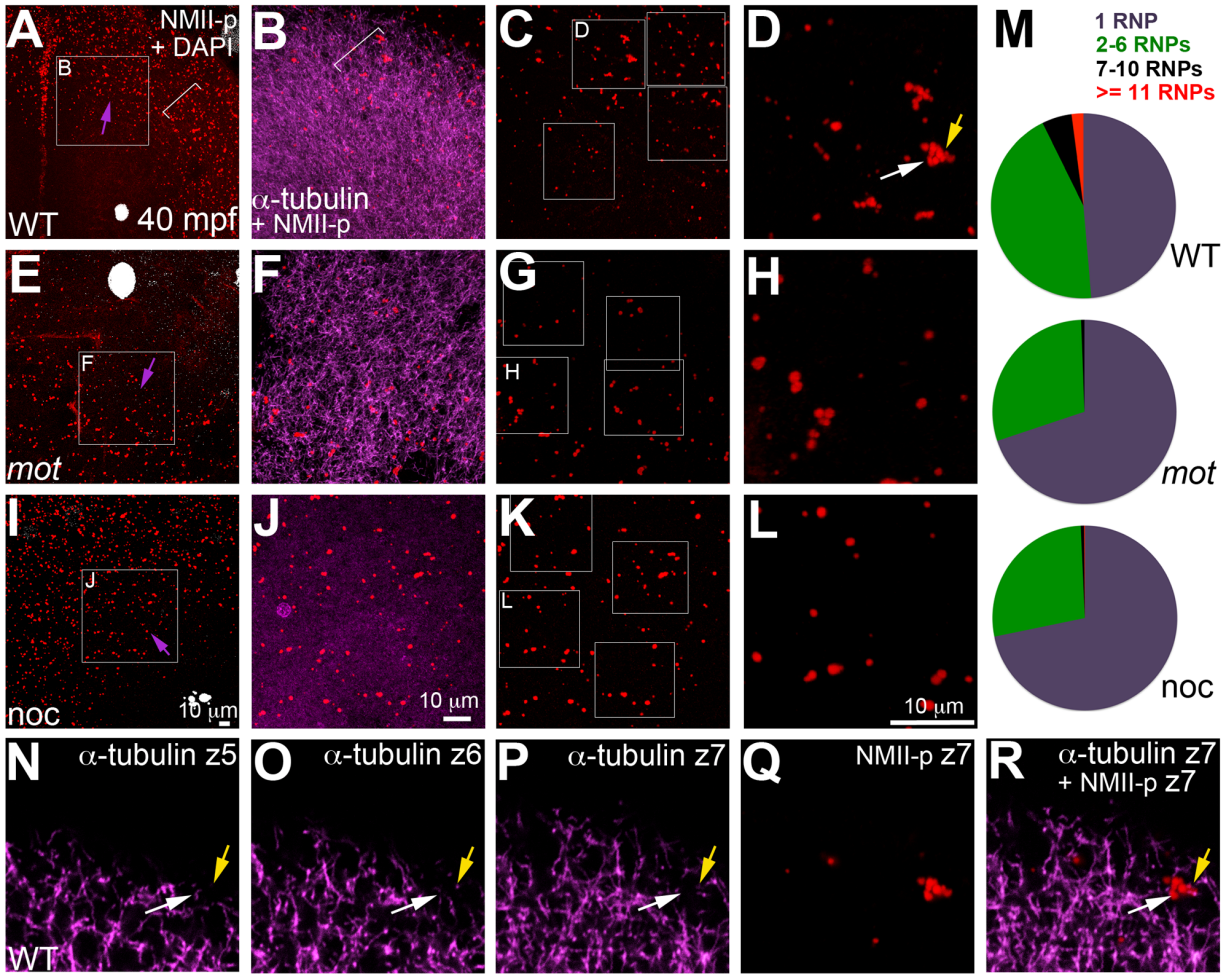
*motley/birc5b* mutants fail to effectively multimerize GP RNPs. Animal views of blastodisc cortex. In wild-type embryos (A–D) the GP RNP aggregation wave (bracket, A) coincides with cortical astral microtubule ends (B, bracket indicates the wavefront within the wave). GP RNP multimerization in wild-type embryos result in large GP RNP aggregates (C, D). In *motley/birc5b* mutants (E–H), cortical microtubules are disorganized (F), and GP RNPs fail to multimerize effectively (G, H). In nocodazole-treated embryos (I–L), cortical microtubules are absent (J) and GP RNP multimerization also fails (K, L). Semi-quantitative analysis of GP RNP aggregation showing similar multimerization failures in *motley/birc5b* and nocodazole-treated embryos compared to wild-type (M). Inset labels in panels A, E, I and C, G, and K indicate ROIs shown at higher magnifications next to the respective panels. Arrows in A, E and I indicate the radial direction, from the center of the blastodisc, along which microtubules in B tend to be oriented in wild-type embryos. (N–R) consecutive confocal sections acquired with a z-step of 0.5 microns: z5 (N, most cortical), z6 (O) and z7 (P, Q, most internal). Yellow arrow shows a single microtubule observed in Z6 (O), Z7 (P) and more internal (not shown) planes but not in z5 (N), which appears associated with an RNP in a multimerized aggregate (Q, R). White arrow indicates a pair of microtubules that appear to converge on a different set of RNPs in the same aggregate (Q,R). The merge panel in R corresponds to microtubules and RNPs for z7 to show the association of microtubule tips with RNP aggregates.

As shown in Figure 5, cortical microtubule tips are in contact with peripheral microfilaments. We next tested the spatial relationship between cortical GP RNPs and microtubule ends. We found that prior to, and during the first 2–3 cell cycles, single and multimerized GP RNPs localized to tips of the monoastral and spindle pole astral microtubules at the cortex (Figure 6D–6F). We had previously postulated that cortical F-actin reorganization facilitates GP RNP multimerization prior to furrow formation [16]. Given the cortical microfilament rearrangement defects in *motley/birc5b*, and the localization of microfilaments and GP RNPs to microtubule tips, we asked whether GP RNP multimerization at the cortex would be affected in *motley/birc5b*. We tested this by comparing the degree of GP RNP aggregation (as determined by the number of GP RNPs that appear to be physically adjoined as multimerized aggregates), in *motley/birc5b* mutants to that in wild-type embryos (Figure 7). As described in the preceding section, in wild-type embryos GP RNPs are found in a band at the periphery of the cortex (Figure 6B; Figure S4E; Figure S6A–S6C) and at the tips of astral microtubules (Figure 6D–6F). Within this band, aggregation occurs such that multimerized GP RNPs are located at an apparent wave front (closer to the blastodisc center) and GP RNP singletons in more peripheral regions (closer to the blastodisc edge) (Figure 7A–7D; [16]). We analyzed GP RNP multimerization semi-quantitatively by dividing the embryo into four quadrants and imaging four random regions of interest (ROIs) within the aggregation wave in each quadrant at 300×. GP RNPs in physical contact with each other were considered as multimeric aggregates. Aggregation analysis of the GP RNPs in wild-type embryos revealed a quantal progression of multimerized GP RNP ranging from single GP RNP to multimeric aggregates of up to 17 GP RNPs (Figure 7C, 7D, 7M). In *motley/birc5b* mutants, the peripheral cortical band of GP RNPs exhibited a significant change in composition with an increase in the numbers of single GP RNP and a decrease in the numbers of multimeric GP RNP aggregates (Figure 7G, 7H, 7M). The largest multimerized GP RNPs in *motley/birc5b* mutants consisted of ∼7 GP RNPs compared to multimeric aggregates of ∼17 GP RNPs found in wild-type embryos (Figure 7M). Microtubule depolymerization by nocodazole treatment decreased GP RNP multimerization to an extent comparable to that seen in *motley/birc5b* mutants (Figure 7K–7M). Thus, Birc5b, and as expected, microtubules are required for multimerization of GP RNPs at the periphery of the blastodisc cortex, prior to their recruitment at the cleavage furrow.

### Birc5b localizes to cortical microtubule ends along with F-actin and germ plasm RNPs during the early embryonic cell cycles

The cortical cytoskeletal and GP RNP multimerization defects in *motley/birc5b* indicate an essential role for cortical microtubules and an additional specific role for Birc5b as a mediator of microtubule-dependent cortical microfilament rearrangements and GP RNP multimerization. This process occurs both prior to (when the sperm monoaster forms) and during early zygotic mitoses (when the spindle pole asters form), prompting us to assay for subcellular localization of Birc5b at the cortex during these early stages.

**Figure 8:**
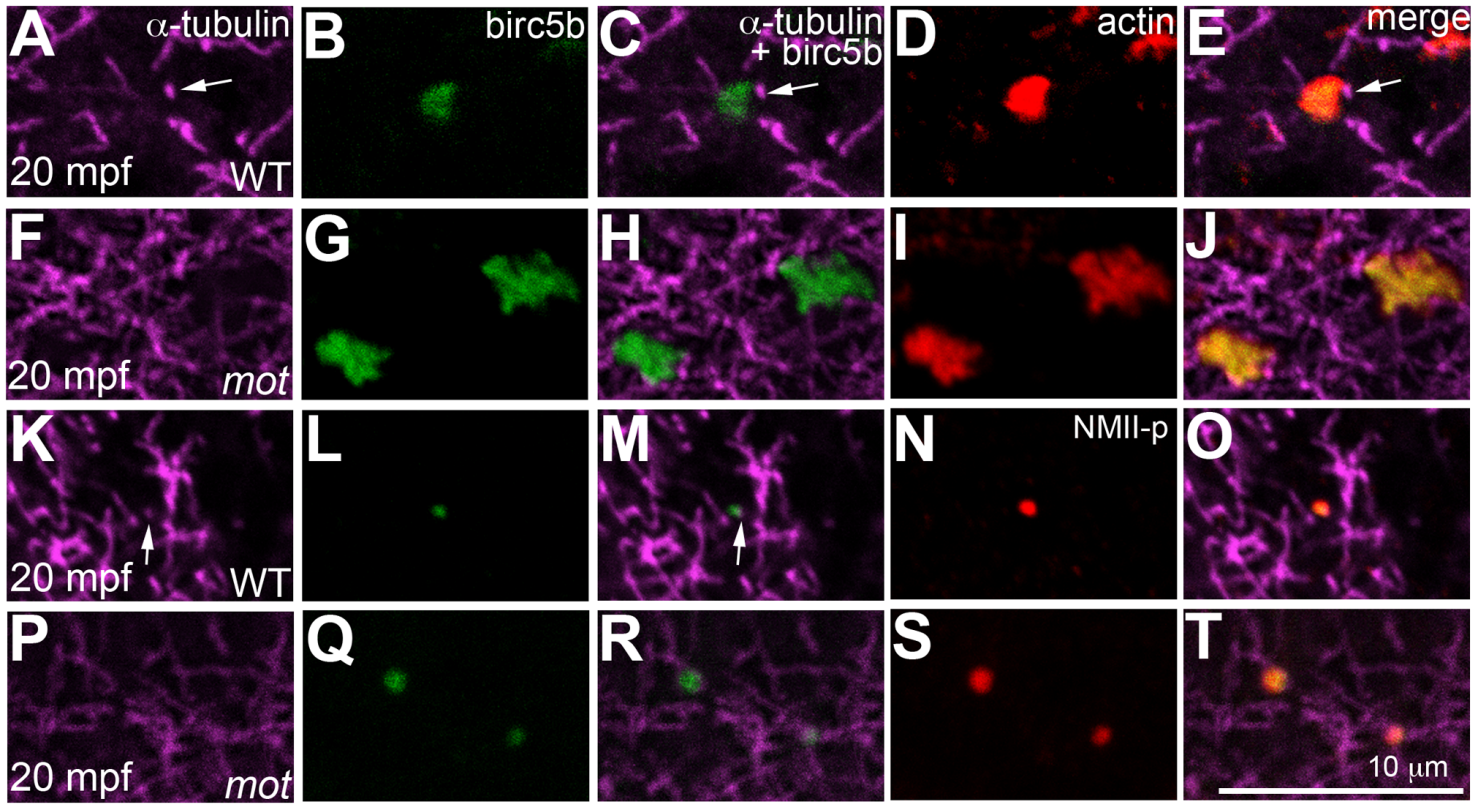
Birc5b localizes to cortical microtubule tips and co-localizes with F-actin and germ plasm RNPs. High magnifications of animal views of blastodisc cortex. In wild-type embryos, Birc5b (B, L) localizes to monoaster microtubule tips (A, C, K, M, arrows) and polymerizing F-actin (D, E). In *motley/birc5b* mutants (F–J) Birc5b co-localizes with polymerizing F-actin (I, J), but as tips of the microtubules are not seen at the cortex (F), co-localization to the microtubule tip is not observed. Birc5b at the microtubule tips (K, M arrow) co-localizes with GP RNPs in wild-type embryos (N, O). In *motley/birc5b* mutants (P–T), microtubule tips are not seen at the cortex (P) but Birc5b continues to localize with GP RNPs (S, T).

In wild-type embryos, Birc5b localized to the tips of cortical sperm monoaster microtubules (Figure 8A–8C, 8K–8M), and co-localized with F-actin seed filaments (Figure 8B, 8D, 8E), and GP RNPs (Figure 8L, 8N, 8O). In *motley/birc5b* mutants, Birc5b continued to co-localize with the F-actin seed filaments (Figure 8G, 8I, 8J) and GP RNPs at the cortex (Figure 8Q, 8S, 8T). Testing colocalization to microtubule tips was precluded by our inability to detect the microtubule tips themselves *in motley/birc5b* mutants (Figure 8F, 8P). However, the lack of peripherally-directed clearing of F-actin seed filaments (Figure 5) and associated GP RNPs (Figure S6) and the failure of GP RNP aggregation in *motley/birc5b* mutants (Figure 7) are consistent with a lack of interaction between Birc5b and associated factors to the tips of growing astral microtubules. Consistent with a role for Birc5b in GP RNP multimerization prior to and during furrow formation, colocalization of Birc5b to GP RNPs in wild-type embryos was maintained during furrow formation but was not observed after the germ plasm was fully compacted at the 4-cell stage (data not shown).

### Germ plasm RNP aggregates are recruited bilaterally to cleavage furrows at the tips of furrow-associated spindle pole astral microtubules

As cleavage furrows form, GP RNPs are recruited to the furrow, forming a rod-like structure at the distal end of the furrow [22], [23], [24]. Given our findings that GP RNPs localize to tips of peripheral astral microtubules prior to (Figure 8K–8O) and during (Figure 6D–6F) furrow formation, we also characterized the association of the GP RNPs to microtubules at the furrow region. Furrow induction occurs when astral microtubules from each side of the bipolar spindle reach the prospective site of cytokinesis at the equatorial cortex [21]. At this incipient furrow, GP RNP aggregates can be observed bound to furrow astral microtubule ends in two rows abutting the furrow center (Figure 6G–6I), likely representing aggregates from each opposing set of astral microtubules that meet at the furrow. This observed bilateral arrangement of GP RNPs support a previously proposed hypothesis that furrow recruitment of GP RNPs involves their gradual gathering near the furrow, enriched by the action of opposing sets of furrow-associated astral microtubules [25].

In summary, our analyses of the zebrafish maternal-effect mutation *motley* identifies it as Birc5b, one of two zebrafish paralogs of the mammalian CPC protein, Survivin/Birc5. Phenotypic and subcellular localization studies suggest a novel function for maternal Birc5b in establishing contact between tips of cortical astral microtubules and cortical F-actin seed filaments and GP RNPs in the early zebrafish embryo. This contact ensures the concerted growth of microtubules and polymerizing microfilaments at the cortex, which results in the reorganization of cortical cytoskeleton to facilitate GP RNP multimerization.

## Discussion

The Chromosomal Passenger Complex (CPC) consisting of AurB, INCENP (Inner Centromere Protein), Borealin/Dasra and Survivin/Bir1/BIRC5 has been ascribed a number of roles during cell division, including chromosome bi-orientation and cytokinesis [2], [3]. Survivin is a member of the Baculoviral Inhibitor of Apoptosis Repeat Containing (BIRC) protein family and contains a single CX_2_CX_16_HX_6_C BIR domain [26]. The BIR domain is an evolutionarily conserved Zn finger fold present in Inhibitor of Apoptosis (IAP) proteins from Baculoviruses to humans [27], [28], [29]. Survivin/BIRC5 is unique amongst CPC and BIRC proteins as it is thought to be directly involved in both cytokinesis and cell survival, though its exclusivity to each process is debated [30].

Here, we show that *birc5b* is largely expressed maternally in zebrafish and that a maternal-effect mutation *motley*, corresponds to *birc5b*. Our characterization of the *motley* mutation reveals maternal functions for *motley/birc5b* during meiosis II completion in oocytes and early embryonic cell divisions, due to its conserved cell cycle-associated CPC activity in zebrafish. Additionally, we uncover a novel role for *motley/birc5b* in the interaction between astral microtubules, F-actin and germ plasm RNPs, which is essential for cytoskeletal rearrangements and GP RNP aggregation immediately after fertilization and during furrow initiation.

Of the two zebrafish paralogs, *birc5b* is expressed exclusively maternally (this study), as opposed to *birc5a*, which is expressed both maternally and zygotically throughout development ([18], this study). These observations are consistent with the observed maternal-effect phenotype of *motley/birc5b*, as well as with morpholino knockdown analysis that show an essential zygotic role for *birc5a*, but not *birc5b*, during late embryogenesis [18], [31], [32]. The defects observed in *motley/birc5b* mutant embryos, despite the presence of endogenous maternal Birc5a protein and our inability to rescue *motley/birc5b* by exogenous maternal expression of Birc5a, indicate that Birc5b has unique maternally-derived functions required during early embryonic cell divisions for GP RNP aggregation. Our analysis is also consistent with previous reports for a requirement for CPC proteins in meiosis in oocytes and sperm of both vertebrates and invertebrates [5], [6], [19], [20], [33], [34], [35], and highlights a dedicated role for the maternal *motley/birc5b* paralog in this process in the zebrafish. Even though both *birc5b* and *birc5a* are expressed during spermatogenesis in the zebrafish (S.N. unpublished data), fertilization rates in crosses with homozygous *motley* mutant males appear unaffected, suggesting that that Birc5b may not have a role during spermatogenesis, or that it functions redundantly with Birc5a in this process. We can not rule out, however, that the mutation results in limited sperm production that is obscured by excess sperm during fertilization, and further studies will be required to address a potential role of this gene during spermatogenesis.

Our analysis in the early embryo relies on antibodies that in western analysis recognize Birc5a and Birc5b forms without cross-reactivity and therefore should be specific to each gene homolog. However, we can not rule out the possibility that these antibodies are cross-reactive in fixed embryos and therefore that labeling signals to detect Bir5b in wild-type and birc5b/motley mutants are not caused by the presence of Birc5a. In spite of this uncertainty, our experiments indicate that Birc5b, but not Birc5a, has a functional role in GP RNP segregation and early embryonic cell division. Recent studies have highlighted the functional overlap and gradual transition between maternal products involved in female meiosis and the early embryonic cell cycles [36], and *birc5b/motley* may constitute an example of intergenerational overlap in gene function, with birc5b/motley acting during maternal meiosis and the early embryonic cycles and birc5a acting at later embryonic stages. Future studies will determine the precise Birc5 forms present in GP RNPs and whether these are associated with other CPC components.

The CPC is present diffusely along chromatin and its centromeric concentration during mitotic metaphase is essential for its function in sister chromatid segregation [2]. The BIR domain of Survivin and several conserved residues within it are required for CPC centromeric concentration [37], [38], [39], [40], [41], [42]. The splice site mutation in the *motley* mutant allele leads to protein mis-translation of Birc5b, truncating its BIR domain and eliminating key conserved residues in the zinc-finger fold. This missing conserved protein domain likely results in the chromosomal segregation defects observed in this mutant both during meiosis and embryonic mitosis. The mitotic chromosome segregation errors in the zebrafish mutant *motley* reflect an evolutionarily conserved role for Birc5b and the CPC during mitosis in the zebrafish embryo.

During anaphase and telophase, CPC proteins localize to the central spindle and overlying equatorial cortex, where their expression precedes the actomyosin assembly required for cytokinesis [2]. A number of studies using separation-of-function alleles have revealed that, in addition to a well-described role in furrow completion [3], CPC function is important for furrow initiation [9], [10], [11]. Our studies confirm these conclusions, since cytokinesis furrows never initiate ingression in *motley/birc5b* mutants. Previous studies have shown that, even with defective DNA segregation, zebrafish embryos undergo normal cytokinesis through signals from asters formed from duplicating centrosomes [21], [43], [44], [45], [46], indicating that the cytokinesis defects observed in *motley* mutants are unlikely caused by prior defects in meiosis or mitosis.

It has been proposed that furrow ingression is triggered by low microtubule density at the cortex, achieved by local bundling of microtubules and/or by separation of astral microtubules [47]. Indeed, a clear boundary free of astral microtubule ends is normally established along the site of furrow initiation in the early zebrafish blastodisc. In *motley/birc5b* mutant embryos during anaphase and telophase, this microtubule-free boundary fails to be established as astral microtubules from each half of the spindle fail to separate distinctly, and are instead found as an interwoven mesh. Furthermore, in *motley/birc5b*, abutting microtubules fail to bundle at the equatorial cortex, as it normally occurs in wild-type embryos. Together with these findings, our observation that Birc5b protein localizes to the tips of bundled microtubules of the incipient furrow at the equatorial cortex suggest that Birc5b may play a direct role in initiating furrow ingression by facilitating low microtubule densities at incipient furrows.

Despite requirements of the CPC in actomyosin contractile ring formation and/or function, very little is known about potential interactions between the CPC components and microfilaments. The striking failure in cortical microfilament reorganization in *motley/birc5b* mutants is the first direct evidence that a member of the CPC is required for actin cytoskeleton rearrangements. In early zebrafish embryos, this cortical microfilament reorganization appears to be essential for GP RNP multimerization, which may facilitate efficient GP RNP recruitment into cytokinesis furrows. The resulting aggregation and subcellular localization of GP RNPs during early embryonic divisions is integral to the selective inheritance of this cell fate determinant at later stages of zebrafish development. Immunoprecipitation analysis has failed to detect a direct interaction between Survivin and F-actin (S.N. unpublished). However, GP RNPs are known to become anchored to the actin cytoskeleton in a variety of systems [48] and one possible scenario is that the association between Survivin and F-actin is mediated by other GP RNP components.

Animal embryos regulate transmission of germ plasm by restricting its localization to specific sites either in the mature egg or in the post-fertilized embryo. In *Drosophila* oocytes, *oskar* mRNA as well as RNPs containing *vasa* and *nanos* are transported during oogenesis along microtubules to the posterior pole of the oocyte, where they become anchored to the actin cortex [13] prior to their incorporation into primordial germ cells at the posterior pole of the embryo [14], [15]. In *Xenopus* the germ plasm is transported during oogenesis to the vegetal pole through association with a specialized cytoplasm called the mitochondrial cloud, resulting in the anchoring of the germ plasm at the vegetal pole cortex. After fertilization, *Xenopus* germ plasm undergoes aggregation at the vegetal pole in a process dependent on microtubules and the kinesin-like protein Xklp1 [49]. In zebrafish oocytes, germ plasm components such as *vasa*, *nanos*, *dazl* also localize to the mitochondrial cloud (Balbiani body), to become associated with the cortex [50]. While some mRNAs, such as *dazl*, maintain their association with the vegetal pole, others acquire a more dispersed pattern and become redistributed to the blastodisc cortex in mature eggs [50]. The mechanism for this early pattern of localization of germ plasm components in zebrafish eggs is currently uncharacterized.

In *Drosophila*, continued localization of *oskar* and *nanos* mRNA, and Oskar and Vasa protein requires a microfilament-dependent anchor [13]. In early zebrafish embryos GP RNPs are initially distributed within a wide band at the periphery of the blastodisc cortex where the microfilaments are arranged in concentric, overlapping rings. Microtubule depolymerization disrupts the cortical microfilaments and GP RNP multimerization. Based on observations of the dynamic changes in the cortical cytoskeleton and germ plasm mRNAs upon pharmacological treatments, it was proposed that cortical astral microtubule ends push microfilaments towards the periphery, a rearrangement that facilitates GP RNP multimerization at the cortical periphery [16]. However, direct demonstration of the cortical arrangement of microtubules and microfilaments and the mechanism by which such cytoskeletal cross-talk facilitates GP RNP aggregation remained to be elucidated.

**Figure 9:**
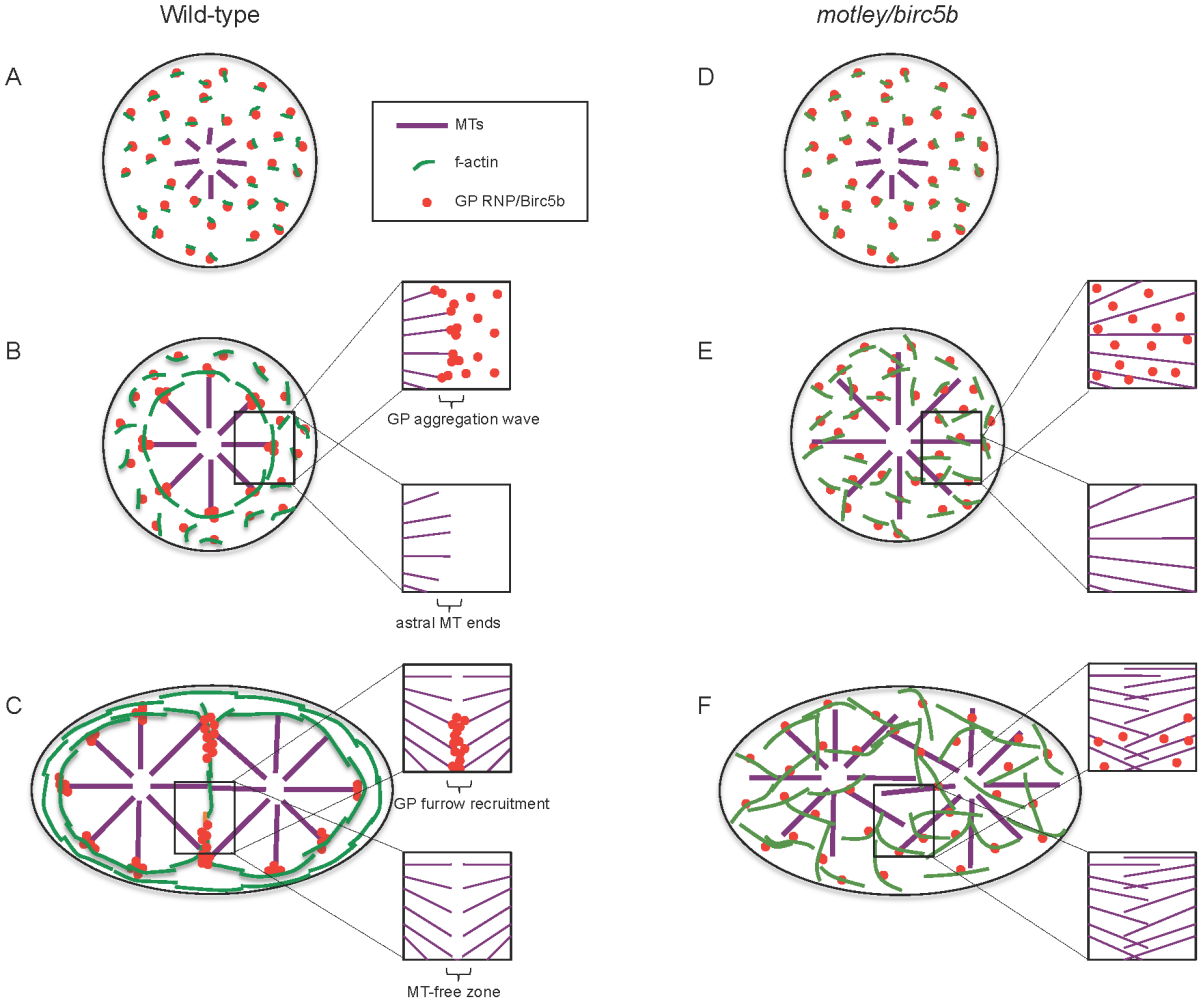
Model of Birc5b function as a potential molecular mediator of microtubule-microfilament interactions facilitating germ plasm RNP multimerization. In wild-type embryos, Birc5b colocalizes with GP RNPs and growing F-actin seed filaments (A). Prior to the first mitosis, this link ensures peripheral movement and circumferential alignment of cortical microfilaments, which we hypothesize provide a substrate that facilitates GP RNP aggregation (B). This results in an aggregating wave of GP RNPs at ends of cortical astral microtubule (B, inserts). During the first embryonic cell division, GP RNP aggregates are recruited bilaterally into forming furrows at the tips contralateral furrow astral microtubule tips (C). In *motley/birc5b* mutants, F-actin seed filaments and GP RNPs associate with Birc5b (D) but microtubule ends fail to contact this complex resulting in defective microfilament reorganization and GP RNP multimerization prior to (E) and during furrow formation (F). Failure to detect microtubule tips at the cortex and the growth of furrow microtubules past each other in *motley/birc5b* mutants are suggestive of a Birc5b-dependent severing function on astral microtubules (compare lower inserts in B–F) both prior to (B, E) and during (C, F) furrow formation. For clarity, inserts in B–F show select components: GP RNPs and microtubules only (upper inserts) and microtubules only (lower inserts). Abbreviations: GP: germ plasm; MT: microtubules.

In this study we show that the ends of astral microtubules at the cortex contact cortical microfilaments, providing support for the hypothesis that expanding astral microtubules push polymerizing microfilaments away from the center of the blastodisc. In *motley/birc5b* mutants, cortical microfilaments are disorganized and GP RNP multimerization is severely reduced, reinforcing previous observations that an intact microfilament network is essential for this process [16]. The present study identifies maternal Birc5b as a molecular mediator of microtubule-microfilament interactions in the early zebrafish embryo. We propose a model wherein Birc5b is present at the blastodisc cortex possibly in a complex with GP RNPs and/or F-actin seed filaments prior to first embryonic mitosis (Figure 9A). As sperm monoaster microtubules reach the cortex they may make contact with Birc5b, which couples the polymerizing f-actin filaments to the tips of peripherally expanding astral microtubules (Figure 9B). This begins the re-positioning of the microfilaments to the cortical periphery where they are required to facilitate GP RNP multimerization (Figure 9B). Microfilament repositioning continues during the first mitosis and is now mediated by spindle pole astral microtubules, which facilitate the ongoing multimerization of GP RNPs during the first 2–3 cleavage cycles (Figure 9C). Multimerized GP RNPs then become enriched at the forming furrow by recruitment at the ends of abutting furrow microtubules (Figure 9C). In *motley/birc5b* mutants, the Birc5b complex with actin and GP RNPs still form and asters appear to grow normally (Figure 9D). However, we hypothesize that mutant Birc5b is unable to associate with the cortical microtubule ends (Figure 9E). In the mutants, this effectively uncouples astral microtubule ends from the polymerizing F-actin seed filaments at the cortex (Figure 9D–9F) and results in the observed defects in microfilament reorganization and RNP multimerization (Figure 9F).

In *motley/birc5b* mutants, we also find that astral microtubules extend along the cortex suggesting that in mutants they may be unable to respond to a cortical signal that would otherwise cause them to undergo dynamic instability and terminate their growth (Figure 9B, 9E). This inference is further supported by the failure of astral microtubules at the incipient cytokinesis furrow in *motley/birc5b* mutants to bundle and terminate growth past their partners from the contralateral side (Figure 9C, 9F). The observation that Birc5b protein localizes to the ends of both astral microtubule tips and bundled cleavage furrow microtubules is consistent with a role for Birc5b in the regulation of microtubule dynamics at both locations in the developing zebrafish embryo.

This study of zebrafish maternal Birc5b provides novel insights into the functions of a conserved CPC protein. Particularly, Birc5b appears to be a key mediator of microtubule-microfilament interactions, a cross-talk that is fundamental to the dynamic cytoskeletal rearrangements facilitating germ plasm subcellular localization prior to and during cytokinesis furrow initiation.

## Materials and Methods

### Ethics statement

All animals were handled in strict accordance with good animal practice as defined by the relevant national and/or local animal welfare bodies, and all animal work was approved by the appropriate committee (University of Wisconsin – Madison assurance number A3368-01).

### Animal husbandry

Wild-type AB, WIK and *motley* mutant fish lines were maintained under standard conditions at 28.5°C. All experiments other than the linkage mapping of *motley* were carried out using embryos from AB fish.

### Positional cloning of *motley*

Homozygous *motley* mutant males were crossed to WIK females to raise F1 fish, which were then in-crossed to obtain the F2 mapping generation. Embryos from F2 females were screened for the syncytial phenotype at 2–4hpf. Genomic DNA from F2 females that produced syncytial mutant clutches was analyzed for segregation of a pan genomic panel of 244 SSLP markers to link and map the *motley* lesion to a genomic locus. The SSLP markers z13363 and z31657 flanked the *motley* locus and z14967 was the closest linked marker on chromosome 23. The *motley* mutation was maintained by crossing homozygous mutant males to heterozygous females.

### *birc5b* cloning and plasmid constructs

Total RNA was isolated from wild-type and *motley* eggs using the TRIZOL reagent (Invitrogen). cDNA was synthesized using an oligodT primer and AMV reverse transcriptase (Promega). *birc5b* alleles from wild-type and *motley* were amplified from cDNA with primers 5′-cagcaatccacacgcaaccagg and 5′- gaagatcaaataagagctctcaaatttttgctagtggc using the Easy-A polymerase (Agilent Technologies). *birc5b* PCR products were ligated into the pGEM-Teasy vector (Promega) for further analyses. Anti-sense *birc5b* WT mRNA for whole mount in situ hybridizations was synthesized from the T7 promoter in pGEM-Teasy after linearizing with PstI. *birc5b::eGFP* fusion construct was created by subcloning the wild-type *birc5b* allele from pGEM-Teasy using primers 5′-GAATTC**atg**tcgaacacagacgttatcgc-3′ and 5′-CTCGAGaataagagctctcaaatttttgctagtgg-3′ that contained EcoRI and XhoI restriction sites respectively. The *eGFP* coding sequence was subcloned from pEGFP-N1 plasmid (GenBank U55762.1) originally from Clontech using primers 5′-CTCGAG**atg**gtgagcaagggc-3′ and 5′-TCTAGA**tta**cttgtacagctcgtccatgcc-3′ that contained XhoI and XbaI restriction sites respectively. Subcloned *birc5b* and *eGFP* were then sequentially cloned in frame into the expression vector pCS2p+. Sense mRNA for rescue experiments was synthesized from the SP6 promoter in pCS2p+ after linearizing with NotI, using the mMessage mMachine kit (Ambion).

### Sequence analysis

All clones were sequenced using BigDye Terminator sequencing and analyzed using 4Peaks and DNAStar Lasergene program suites. Protein sequences were compared using ClustalX and the phylogenetic tree was visualized using TreeView. We cloned Birc5a and Birc5b as proteins of 190 and 144 amino acids respectively, each from contiguous maternal transcripts. This corresponds to the zebrafish sequence information available at http://vega.sanger.ac.uk/index.html. However, the sequence information at NCBI lacks the first exon for both Birc5a (GI:68085121) and Birc5b (GI:76780231), listing the proteins as containing 142 and 128 amino acids respectively.

### In vitro oocyte culture and rescue injections

The detailed experimental procedure for injecting and culturing stage IV zebrafish oocytes is available elsewhere [51], [52]. Briefly, homozygous *motley* and AB adult females were purged of mature eggs by natural pair matings. Day 8 or 9 post purging, immature stage IV oocytes were isolated and injected with ∼200 pg of *birc5b::eGFP* or *birc5a::eGFP* mRNA. GFP-expressing mature eggs were manually defolliculated and fixed for meiosis rescue or in vitro fertilized and imaged or fixed for post-fertilization rescue analysis. For zygotic rescues ∼200 pg of *birc5b::eGFP* mRNA was injected into 1-cell embryos and GFP-expressing embryos were imaged and fixed between 2–4hpf.

### Western blotting

∼100–500 wild-type or *motley/birc5b* embryos or eggs were collected and lysed in RIPA buffer on ice using a small syringe after discarding all the embryo medium. Lysates were centrifuged at 13000 rpm for 3–4 mins at 4°C to settle debris and protein concentration was determined. 50–200 µg of protein was loaded onto precast 4–15% TGX gels (BioRad) and blotted onto PVDF membranes for 10 hours at 4°C. Membranes were blotted with 1∶100 anti-Survivin (sc-17779, Santa Cruz Biotechnology Inc) or 1∶500 anti-Survivin-BIR (Unconjugated, Cell Signaling Technology 2808). Membranes were developed using the Fast Western Blot Kit SuperSignal (ThermoScientific). Anti-Survivin-BIR antibodies were developed against conserved BIR aminoacid sequence centered on human Cys60 (Cell Signaling Technology), whose corresponding aminoacid in zebrafish Birc5b is located 15 aminoacids upstream of the site of the mutation in the Motley/Birc5b product.

### Fluorescent immunolabeling

Wild-type and *motley/birc5b* mutant embryos were obtained by in vitro fertilization to synchronize the clutches for all experiments. Embryos were fixed with a paraformaldehyde-glutaraldehyde fixative and immunolabeled as described previously [24]. Primary antibodies used were Mouse anti-α-Tubulin (1∶2500, Sigma T5168), Rabbit anti-β-catenin (1∶1000, Sigma C2206), Rabbit anti-phospho-Myosin Light Chain 2 (1∶50, Cell Signaling Technology 3671L), Rabbit anti-actin (1∶100, Sigma A2066), Rabbit anti-AurB (1∶100, [21]) and Rabbit anti-Survivin Alexa 488 (herein anti-Survivin-BIR, same as used for western analysis but is conjugated; 1∶25, Cell Signaling Technology 2810). Fluorescent secondary antibodies Goat anti-Mouse-Cy5 (Jackson ImmunoResearch Labs (JIL) 115-175-003), Goat anti-Rabbit-Alexa 488 (Molecular Probes A-11008), Goat anti-Rabbit-Cy3 (JIL 111-165-144) and Goat anti-Mouse-Cy3 (JIL 115-165-062) were used at 1∶100. For triple immunolabeling, anti-Survivin-BIR was added after the secondary antibody wash and incubated overnight at 4°C prior to DAPI staining. All immunolabeled embryos were semi-flat mounted for animal views of the blastodisc. Images were obtained using a Zeiss LSM510 confocal microscope and analyzed using ImageJ.

### In situ hybridization

Embryos were fixed in 4% paraformaldehyde for 12 hrs at room temperature, dechorionated and transferred into 100% Methanol at −20°C overnight. Rehydrated embryos were hybridized with antisense *birc5b* overnight at 65°C. Whole mount in situs were developed using an anti-DIG alkaline phosphatase antibody, followed by NBT-BCIP color reaction. Fluorescent in situs were incubated with an anti-DIG-POD antibody (Roche Applied Science) and developed using the Tyramide signal amplification kit (Invitrogen). For immunolabeling after fluorescent in situ hybridization, embryos were washed in PBS-Triton after the Tyramide reaction, deyolked and blocked in antibody block prior to addition of the Rabbit-anti-phospho Myosin Light Chain 2.

### Semi-quantitative analysis of germ plasm RNP multimerization

Embryos immunolabeled at ∼40mpf were divided into 4 quadrants and a Region of Interest (ROI) away from the furrow was imaged in each quadrant at 40×. The location of 40× ROIs was chosen such that it encompassed the RNP aggregation wave front, where multimerization would be most evident. Within each 40× ROI, 4 random ROIs were imaged using a 100× objective and a 3× digital zoom (300× ROIs, 16 per embryo). RNPs from 5 embryos each of WT, *motley/birc5b* and nocodazole treated were pooled for the pie charts. The number of GP RNPs that were directly adjoined was determined in the 300× ROIs using the Cell Counter plugin from ImageJ. This number was used as a semi-quantitative measure of GP RNP multimerization.

## Supporting Information

Figure S1 Cell division defects in the maternal-effect mutants *motley^p1aiue^* and *p4anua* and identification of *motley^p1aiue^* as *birc5b*. (A–H) Furrow formation defect in *motley* (A,B,E,F) and *p4anua* (C,D,G,H) mutants. During the second cell cycle, two intersecting mature cleavage furrows are seen in wild-type embryos (A), and reiterative cytokinesis results in a cellularized blastoderm with distinct nuclei as seen by β-catenin and DAPI labeling (B). During the second cell cycle, the appearance of furrows in *motley* (C) is due to spreading astral microtubules that never mature into furrows (D). DNA segregation errors in *motley* manifest as unevenly distributed DNA patches (D). During the second cell cycle, compared to the wild-type embryo (E, F), mature cleavage furrows are absent in *p4anua* mutants (G, H). (I) *birc5a* is maternally present and is expressed during all stages of embryonic development, while *birc5b* transcript is not detectable beyond 24 hpf. β-actin was used as a control transcript which is expressed constitutively during development (200 bp band in all lanes). (J,K) Whole mount in situ hybridizations for *birc5b* show ubiquitous maternal expression in wild-type (J) and *motley* embryos (K). (L) TreeView rendering of the ClustalX alignment of BIR proteins from several representative species indicate a trichotomy (black, green and pink groupings) in the BIR family and group zebrafish Birc5a and Birc5b with homologous Birc5 proteins. (A–H, J, K) are animal views of blastodiscs.
(TIF)

Figure S2 Birc5b::GFP expression in cultured oocytes and late rescue of *motley* cytokinesis defects by wild-type Birc5b::GFP mRNA injected post-fertilization. (A–F) Expression of Birc5b::GFP through mRNA injection into in vitro matured oocytes. Immature germinal vesicle containing stage IV oocytes from homozygous *motley* females express protein from injected *birc5b::eGFP* mRNA 1 hour post injection (hpi) (A–C). Germinal vesicle dissolution and oocyte clearing occur normally in Birc5b::eGFP-expressing *motley* oocytes (D–F). (G–K) Partial rescue of cytokinesis defects in *motley* mutants by expression of Birc5b::GFP through mRNA injection into 1-cell embryos. *motley* mutant embryos injected with *birc5b::eGFP* at the 1-cell stage are Birc5b::eGFP-positive at 2 hpf and exhibit several cleavage furrows (G–I) like wild-type embryos (K), although blastomeres in mutants are larger due to the lag in functional rescue through injection at the 1-cell stage. Uninjected sibling *motley* mutants do not exhibit any furrows (J). (L–O) Rescue of midbody formation defect in *motley* mutants by expression of Birc5b::GFP through mRNA injection into 1-cell embryos. At 4 hpf, midbodies are seen in wild-type embryos (L, arrows), which are never seen in *motley* mutants (M). In *motley* mutants injected with *birc5b::eGFP* at the 1-cell stage, midbodies are seen by 4 hpf (N, O, arrows). Cells in the panels shown (L–O) are at slightly different stages in the cell cycle (shown by different sizes of asters) due to asynchronicity between embryos and embryonic regions characteristic of these stages [53], [54]. However, midbody structures in the early embryo are stable through multiple cell cycles ([21]; our own observations), allowing a comparison between the various conditions.
(TIF)

Figure S3 Birc5a and Birc5b protein are both expressed maternally, but are functionally non-redundant. (A) Western blot of whole protein lysates from 20 mpa wild-type eggs (lane 1 and 2), and 40 mpf wild-type (lane 3) and *motley* embryos (lane 4). Anti-Survivin detects an ∼25 KDa protein (lane 1) that is unaffected in *motley* mutants (data not shown), consistent with this antibody recognizing Birc5a (190 aa, predicted MW 22 kDa). Anti-Survivin-BIR detects a doublet of ∼15 KDa protein in wild-type lysates (lanes 2, 3), which is affected in *motley* mutants (lane 4: lower band missing, upper band with reduced intensity). The mutant product encoded by the mutant *motley* allele is predicted to include the first 79 aa of the normal protein plus 32 novel aminoacids encoded by intronic sequence (111 aa total, MW ∼13 kDa). Because aminoacid composition can affect protein mobility, determination of the precise identity of each band will require protein analysis. However, the data is consistent with anti-Survivin recognizing Birc5a and anti-Survivin-BIR recognizing Birc5b, and a lack of cross-reactivity between these two antibodies and their products. Total protein from each lysate loaded in each lane is indicated in µg. (B–J) Expression of Birc5a::GFP does not rescue the *motley/birc5b* cytokinesis phenotype. Stage IV oocytes from *motley/*birc5b mutant females were injected with *birc5a::GFP* mRNA and in vitro matured into eggs under the same conditions that allowed rescue through expression of *birc5b::GFP* mRNA. Injected oocytes expressed Birc5a::GFP at ∼1hpi and matured into eggs (data not shown). Embryos derived from such Birc5a::GFP-expressing eggs activated normally upon contact with water following in vitro fertilization (B–D) but did not undergo cytokinesis (E–G). Labeling for α-tubulin and DAPI at a time equivalent to the 4-cell stage confirmed that such Birc5a::GFP-expressing, non-cleaving *motley/birc5b* embryos were indeed fertilized (H–J, note four nuclei showing normal karyokinesis).
(TIF)

Figure S4 Germ plasm RNPs localize onto cortical microfilaments at the blastodisc periphery. Animal views of blastodiscs; D–F are higher magnifications of area indicated in C, rotated 90° counterclockwise. Fluorescent in situ hybridization for the germ plasm mRNA *nanos* (B, E), together with immunolabeling for f-actin (A, D) shows that RNPs labeled with *nanos* co-localize with microfilaments arranged in concentric rings at the cortical periphery (C, F).
(TIF)

Figure S5 Germ plasm mRNPs are labeled by anti-human phosphorylated non-muscle myosin II antibody. Animal views of 2-cell stage embryos; immunolabelings (A–C), fluorescent in situ hybridizations for *nanos* (D, M), *dnd* (E, N) and *vasa* (F, O), combined with immunolabeling for NMII-p (G–I, P–R). (M–U) Higher magnifications of boxed areas in (J–L). NMII-p labeling recapitulates the known patterns of germ plasm mRNA localization to the distal furrows and peripheral cortex in wild-type embryos at ∼45mpf (A–C). *nanos*, *dnd* and *vasa* mRNAs label germ plasm aggregates at the cortical periphery and at the distal ends of the cleavage furrow (D–F, M–O). NMII-p expression overlaps with *nanos* (J, S), *dnd* (K, T) and *vasa* (L, U) both at the outlying cortex and the distal cleavage furrow. (S1) Higher magnification view of an example panel used for the quantitation of colocalization shown in (V), from the image in (S). (V) Pooled counts of observed particles (singletons and multimerized, n = 349) indicate that 76% of particles show both NMII-p and GP RNA labeling (yellow), 19% exhibit only NMII-p labeling (red) and 5% only GP RNA labeling (green). Due to the significantly higher sensitivity of the anti-NMII-p immunofluorescence labeling in comparison to the FISH technique to detect GP RNAs, it is likely that a significant fraction, if not most, of the 19% of particles that exhibit only NMII-p labeling also contain undetected GP RNAs. While we can not rule out that at these stages a minority of particles contain NMII-p without GP RNAs, the observation that less than 5% of particles apparently containing GP RNAs are not labeled with the anti-NMII-p antibody indicates that NMII-p is a reliable marker for GP RNPs.
(TIF)

Figure S6 The cortical band of germ plasm RNPs fails to undergo a peripherally-directed compression in *motley/birc5b* mutants. Animal views of blastodisc cortex, immunolabeled with anti-NMII-p antibody. (A–F) Segregation of GP RNPs during the first two cycles. In wild-type embryos at 20mpf, the center of the blastodisc cortex is GP RNP-free as the RNPs are located in a broad peripheral band (A). As development proceeds, the blastodisc center remains free of GP RNPs and the peripheral band becomes compressed from the center outwards as the central GP RNP-free zone expands (B, C). In *motley/birc5b* mutants at 20mpf, the center of the blastodisc is GP RNP free and the RNPs are in a broad peripheral band as in wild-type embryos (D). However, the peripheral GP RNP band fails to further compress noticeably during development (E, F). Yellow double-headed arrows represent GP RNP-free zone in the blastodisc center, which does not expand in *motley* mutants. White double-headed arrows represent peripheral compression of GP RNP band in wild-type embryos, which does not occur effectively in motley/birc5b. (G–I) In *motley* mutants at a stage coincident with observed GP RNP segregation defects, F-actin (H,I) forms large bundles randomly crisscrossing the blastodisc, instead of circumferential bundles as observed in wild-type (Figure 5B, 5C, 5H; [16]). Such randomly oriented bundles also form in embryos after inhibition of microtubule polymerization [16] and may be related to F-actin gelation observed in early embryonic extracts [55]. DAPI labeling in (G,I) shows center of blastodisc; astral microtubules in G are not clearly visible as this time point coincides with the cyclical disassembly of this structure after reaching the cortex [16], [56]. (J,K) In situ hybridizations to detect germ plasm RNAs *nanos* (J) and *dead end* (K) in *motley* mutant embryos, showing defects in germ plasm segregation similar to those observed when visualizing GP RNPs with anti-NMII-p antibodies (compare to wild-type in Figure S5D, S5E; GP RNP recruitment in wild-type furrows can be detected as early as 30 mpf [16]).
(TIF)
