# Supplementary figures and images for: The Chromosomal Passenger Protein Birc5b Organizes Microfilaments and Germ Plasm in the Zebrafish Embryo
Source: PLoS Genet. 2013 Apr 18;9(4):e1003448. doi: 10.1371/journal.pgen.1003448 (PMC3630083; doi:10.1371/journal.pgen.1003448)

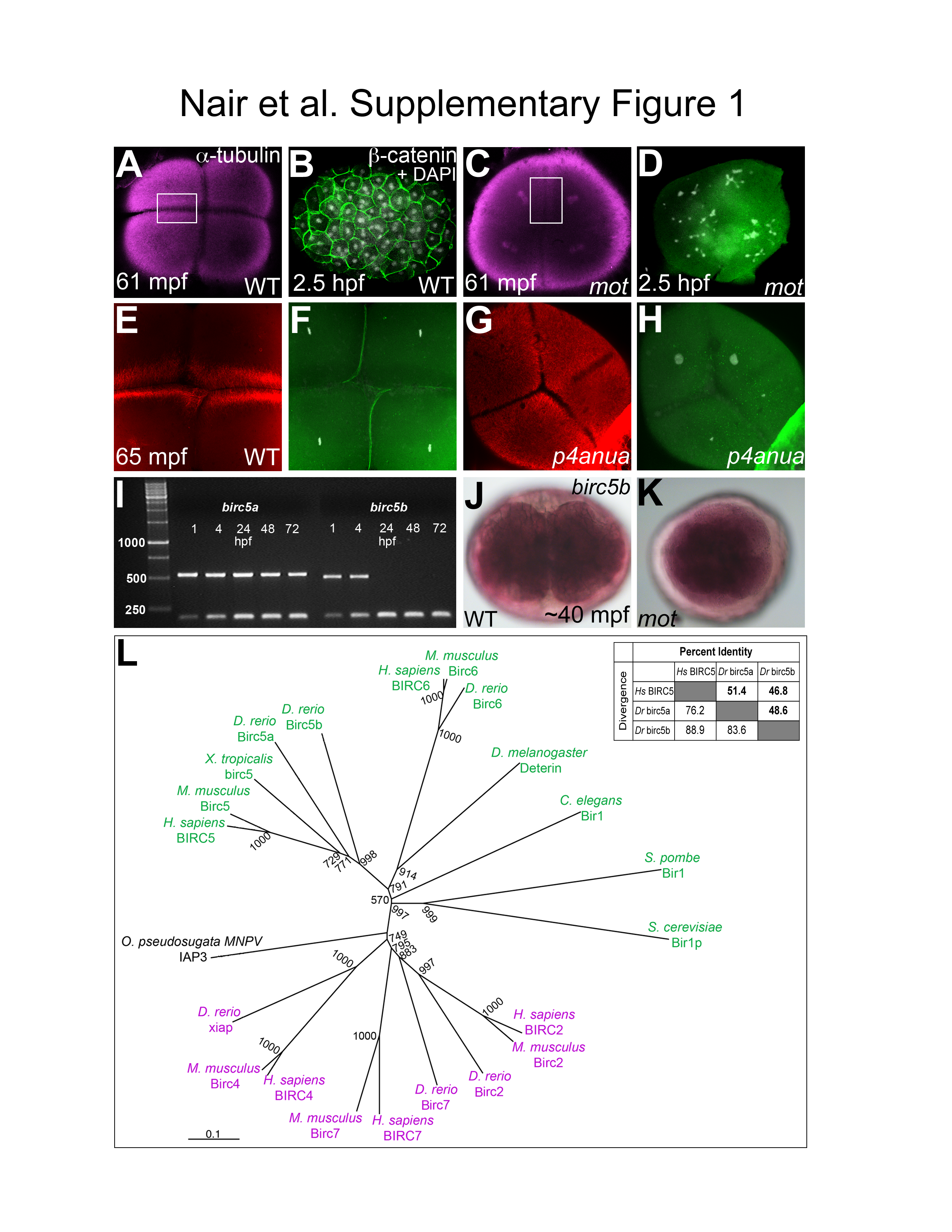

Supplement: Figure S1 — Cell division defects in the maternal-effect mutants motleyp1aiue and p4anua and identification of motleyp1aiue as birc5b. (A–H) Furrow formation defect in motley (A,B,E,F) and p4anua (C,D,G,H) mutants. During the second cell cycle, two intersecting mature cleavage furrows are seen in wild-type embryos (A), and reiterative cytokinesis results in a cellularized blastoderm with distinct nuclei as seen by β-catenin and DAPI labeling (B). During the second cell cycle, the appearance of furrows in motley (C) is due to spreading astral microtubules that never mature into furrows (D). DNA segregation errors in motley manifest as unevenly distributed DNA patches (D). During the second cell cycle, compared to the wild-type embryo (E, F), mature cleavage furrows are absent in p4anua mutants (G, H). (I) birc5a is maternally present and is expressed during all stages of embryonic development, while birc5b transcript is not detectable beyond 24 hpf. β-actin was used as a control transcript which is expressed constitutively during development (200 bp band in all lanes). (J,K) Whole mount in situ hybridizations for birc5b show ubiquitous maternal expression in wild-type (J) and motley embryos (K). (L) TreeView rendering of the ClustalX alignment of BIR proteins from several representative species indicate a trichotomy (black, green and pink groupings) in the BIR family and group zebrafish Birc5a and Birc5b with homologous Birc5 proteins. (A–H, J, K) are animal views of blastodiscs. (TIF) [file pgen.1003448.s001.tif]

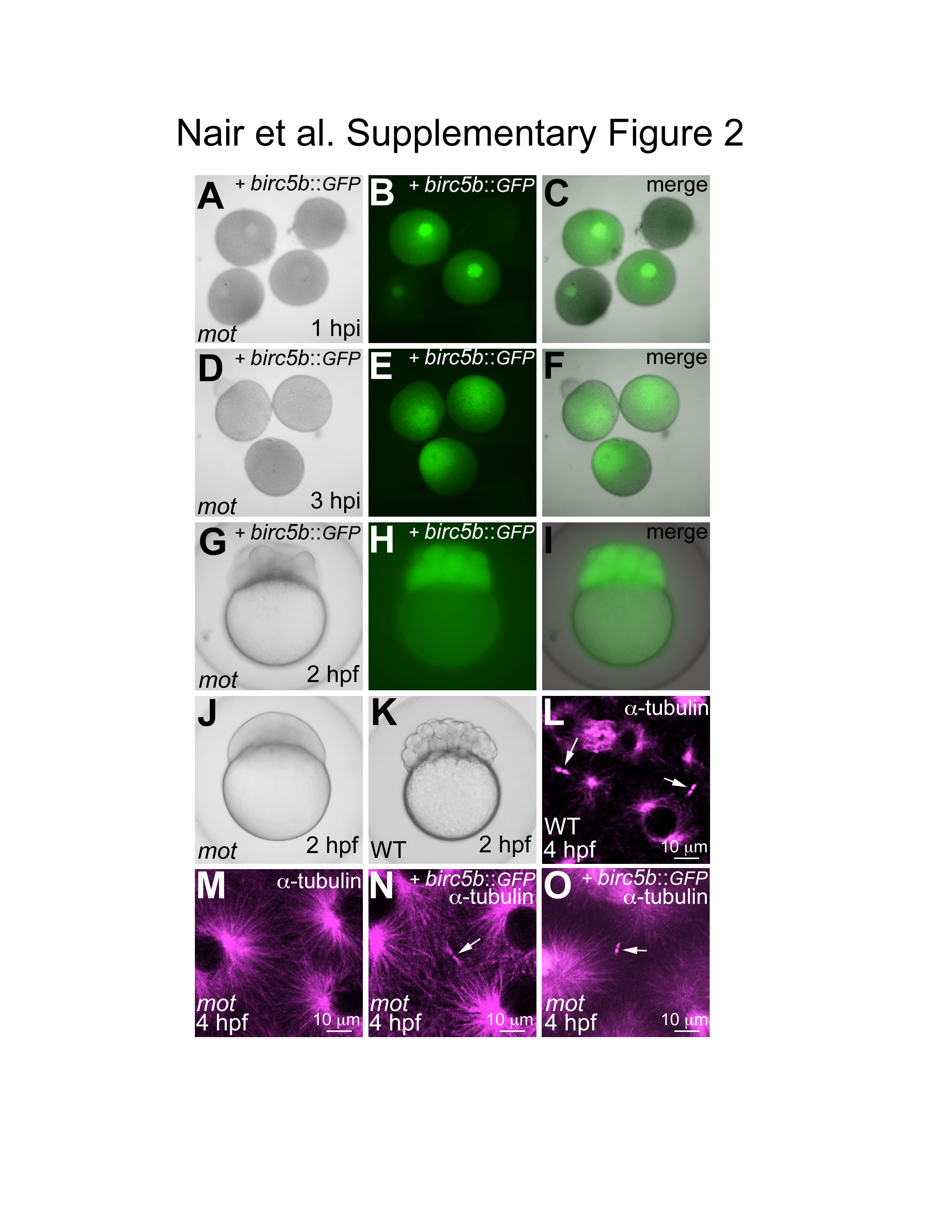

Supplement: Figure S2 — Birc5b::GFP expression in cultured oocytes and late rescue of motley cytokinesis defects by wild-type Birc5b::GFP mRNA injected post-fertilization. (A–F) Expression of Birc5b::GFP through mRNA injection into in vitro matured oocytes. Immature germinal vesicle containing stage IV oocytes from homozygous motley females express protein from injected birc5b::eGFP mRNA 1 hour post injection (hpi) (A–C). Germinal vesicle dissolution and oocyte clearing occur normally in Birc5b::eGFP-expressing motley oocytes (D–F). (G–K) Partial rescue of cytokinesis defects in motley mutants by expression of Birc5b::GFP through mRNA injection into 1-cell embryos. motley mutant embryos injected with birc5b::eGFP at the 1-cell stage are Birc5b::eGFP-positive at 2 hpf and exhibit several cleavage furrows (G–I) like wild-type embryos (K), although blastomeres in mutants are larger due to the lag in functional rescue through injection at the 1-cell stage. Uninjected sibling motley mutants do not exhibit any furrows (J). (L–O) Rescue of midbody formation defect in motley mutants by expression of Birc5b::GFP through mRNA injection into 1-cell embryos. At 4 hpf, midbodies are seen in wild-type embryos (L, arrows), which are never seen in motley mutants (M). In motley mutants injected with birc5b::eGFP at the 1-cell stage, midbodies are seen by 4 hpf (N, O, arrows). Cells in the panels shown (L–O) are at slightly different stages in the cell cycle (shown by different sizes of asters) due to asynchronicity between embryos and embryonic regions characteristic of these stages [53], [54]. However, midbody structures in the early embryo are stable through multiple cell cycles ([21]; our own observations), allowing a comparison between the various conditions. (TIF) [file pgen.1003448.s002.tif]

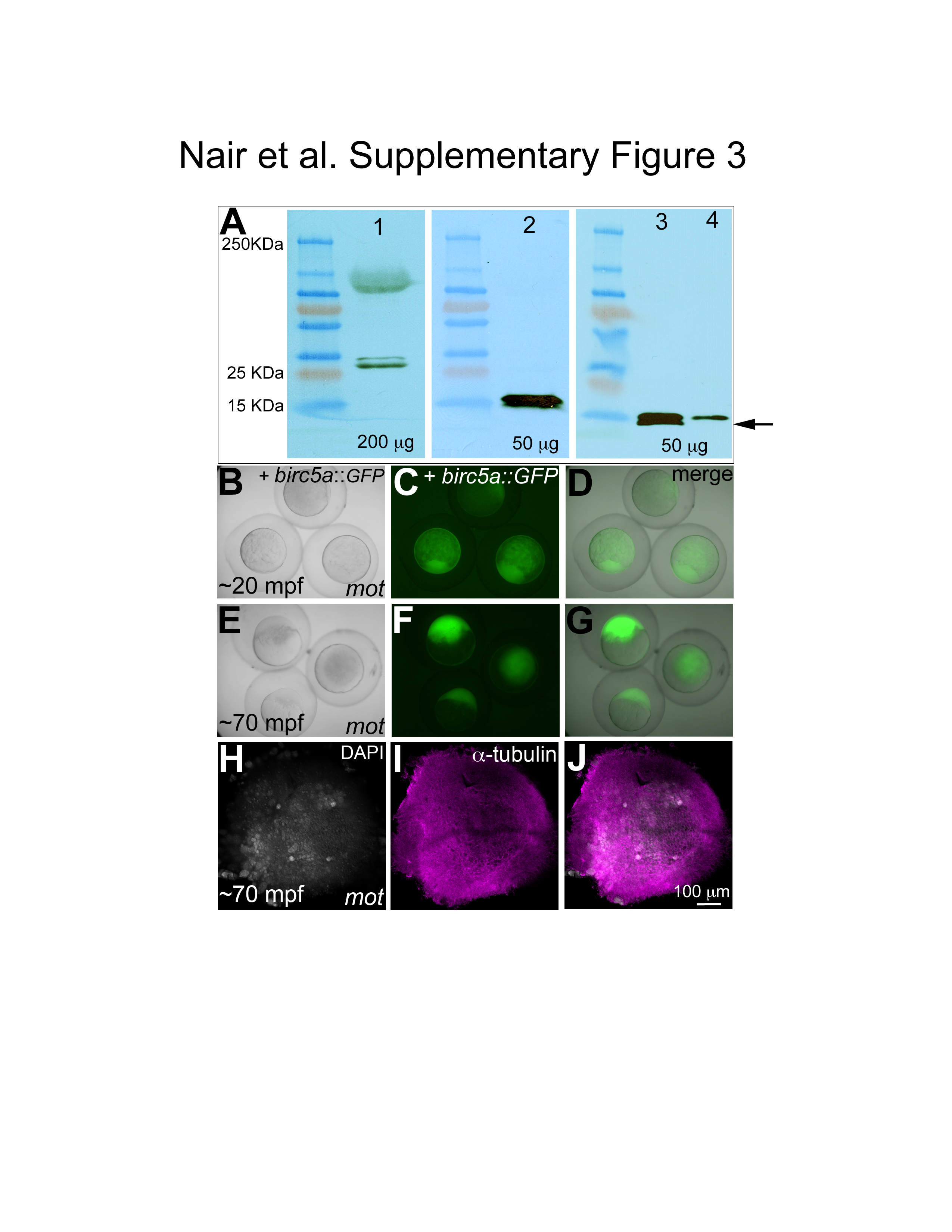

Supplement: Figure S3 — Birc5a and Birc5b protein are both expressed maternally, but are functionally non-redundant. (A) Western blot of whole protein lysates from 20 mpa wild-type eggs (lane 1 and 2), and 40 mpf wild-type (lane 3) and motley embryos (lane 4). Anti-Survivin detects an ∼25 KDa protein (lane 1) that is unaffected in motley mutants (data not shown), consistent with this antibody recognizing Birc5a (190 aa, predicted MW 22 kDa). Anti-Survivin-BIR detects a doublet of ∼15 KDa protein in wild-type lysates (lanes 2, 3), which is affected in motley mutants (lane 4: lower band missing, upper band with reduced intensity). The mutant product encoded by the mutant motley allele is predicted to include the first 79 aa of the normal protein plus 32 novel aminoacids encoded by intronic sequence (111 aa total, MW ∼13 kDa). Because aminoacid composition can affect protein mobility, determination of the precise identity of each band will require protein analysis. However, the data is consistent with anti-Survivin recognizing Birc5a and anti-Survivin-BIR recognizing Birc5b, and a lack of cross-reactivity between these two antibodies and their products. Total protein from each lysate loaded in each lane is indicated in µg. (B–J) Expression of Birc5a::GFP does not rescue the motley/birc5b cytokinesis phenotype. Stage IV oocytes from motley/birc5b mutant females were injected with birc5a::GFP mRNA and in vitro matured into eggs under the same conditions that allowed rescue through expression of birc5b::GFP mRNA. Injected oocytes expressed Birc5a::GFP at ∼1hpi and matured into eggs (data not shown). Embryos derived from such Birc5a::GFP-expressing eggs activated normally upon contact with water following in vitro fertilization (B–D) but did not undergo cytokinesis (E–G). Labeling for α-tubulin and DAPI at a time equivalent to the 4-cell stage confirmed that such Birc5a::GFP-expressing, non-cleaving motley/birc5b embryos were indeed fertilized (H–J, note four nuclei showing normal karyokinesis). [file pgen.1003448.s003.tif]

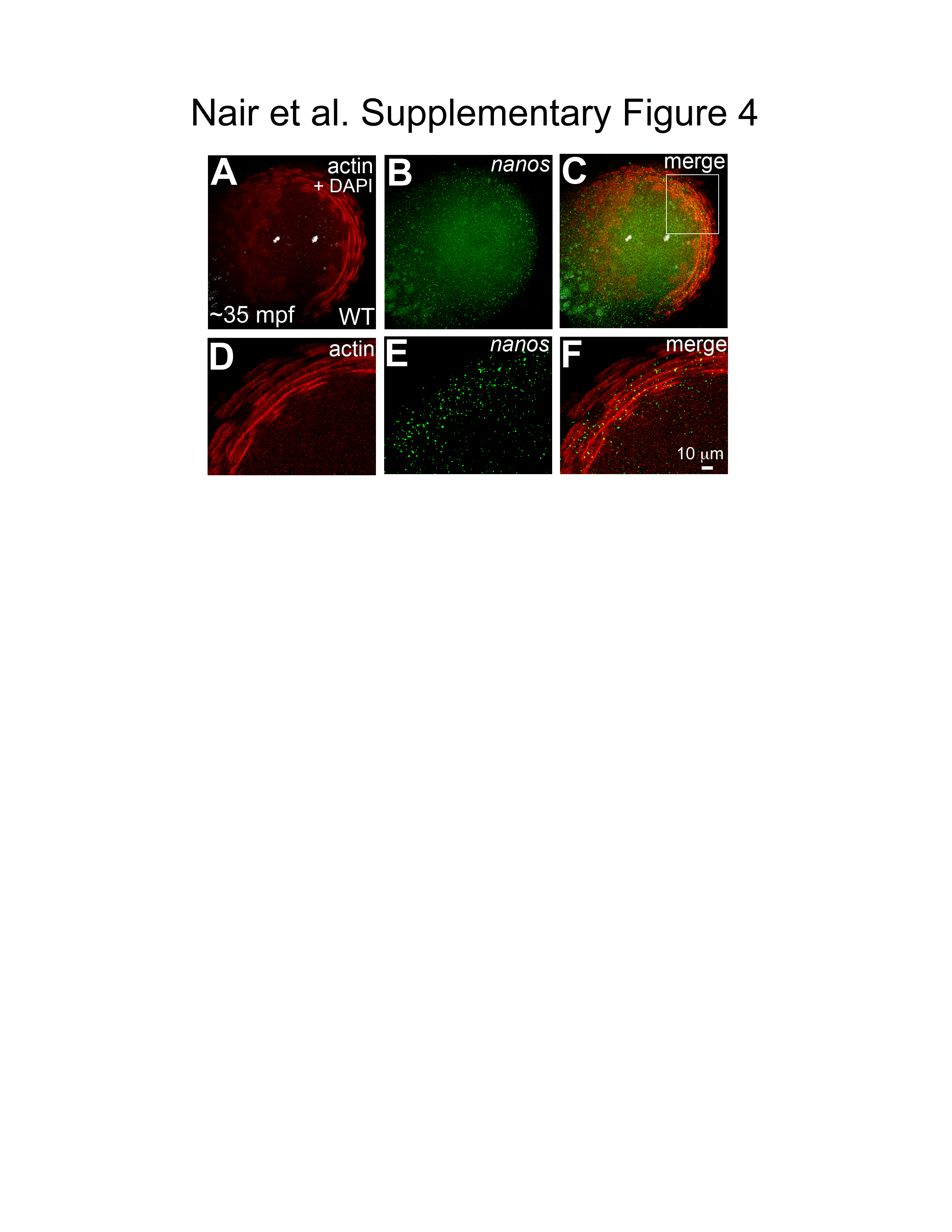

Supplement: Figure S4 — Germ plasm RNPs localize onto cortical microfilaments at the blastodisc periphery. Animal views of blastodiscs; D–F are higher magnifications of area indicated in C, rotated 90° counterclockwise. Fluorescent in situ hybridization for the germ plasm mRNA nanos (B, E), together with immunolabeling for f-actin (A, D) shows that RNPs labeled with nanos co-localize with microfilaments arranged in concentric rings at the cortical periphery (C, F). (TIF) [file pgen.1003448.s004.tif]

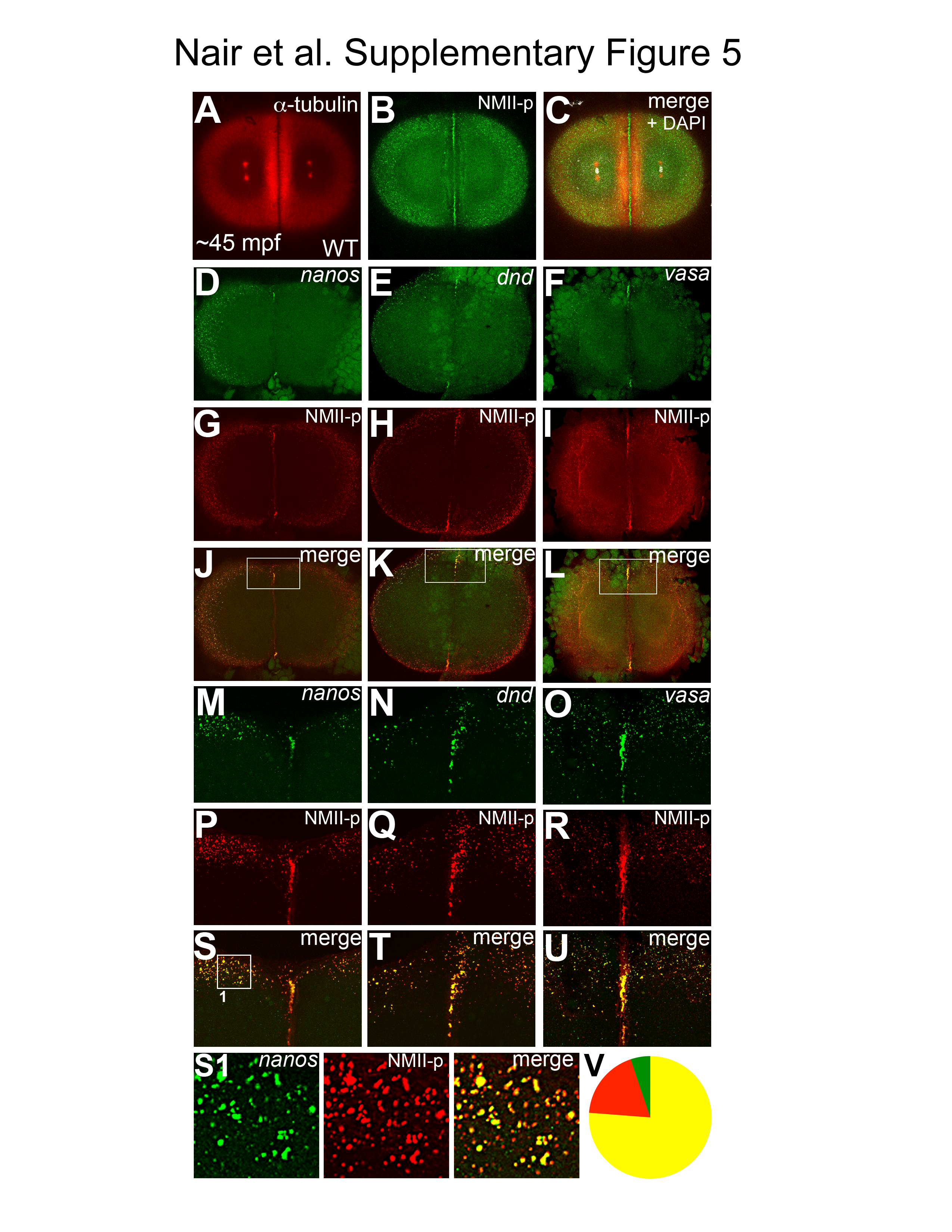

Supplement: Figure S5 — Germ plasm mRNPs are labeled by anti-human phosphorylated non-muscle myosin II antibody. Animal views of 2-cell stage embryos; immunolabelings (A–C), fluorescent in situ hybridizations for nanos (D, M), dnd (E, N) and vasa (F, O), combined with immunolabeling for NMII-p (G–I, P–R). (M–U) Higher magnifications of boxed areas in (J–L). NMII-p labeling recapitulates the known patterns of germ plasm mRNA localization to the distal furrows and peripheral cortex in wild-type embryos at ∼45mpf (A–C). nanos, dnd and vasa mRNAs label germ plasm aggregates at the cortical periphery and at the distal ends of the cleavage furrow (D–F, M–O). NMII-p expression overlaps with nanos (J, S), dnd (K, T) and vasa (L, U) both at the outlying cortex and the distal cleavage furrow. (S1) Higher magnification view of an example panel used for the quantitation of colocalization shown in (V), from the image in (S). (V) Pooled counts of observed particles (singletons and multimerized, n = 349) indicate that 76% of particles show both NMII-p and GP RNA labeling (yellow), 19% exhibit only NMII-p labeling (red) and 5% only GP RNA labeling (green). Due to the significantly higher sensitivity of the anti-NMII-p immunofluorescence labeling in comparison to the FISH technique to detect GP RNAs, it is likely that a significant fraction, if not most, of the 19% of particles that exhibit only NMII-p labeling also contain undetected GP RNAs. While we can not rule out that at these stages a minority of particles contain NMII-p without GP RNAs, the observation that less than 5% of particles apparently containing GP RNAs are not labeled with the anti-NMII-p antibody indicates that NMII-p is a reliable marker for GP RNPs. (TIF) [file pgen.1003448.s005.tif]

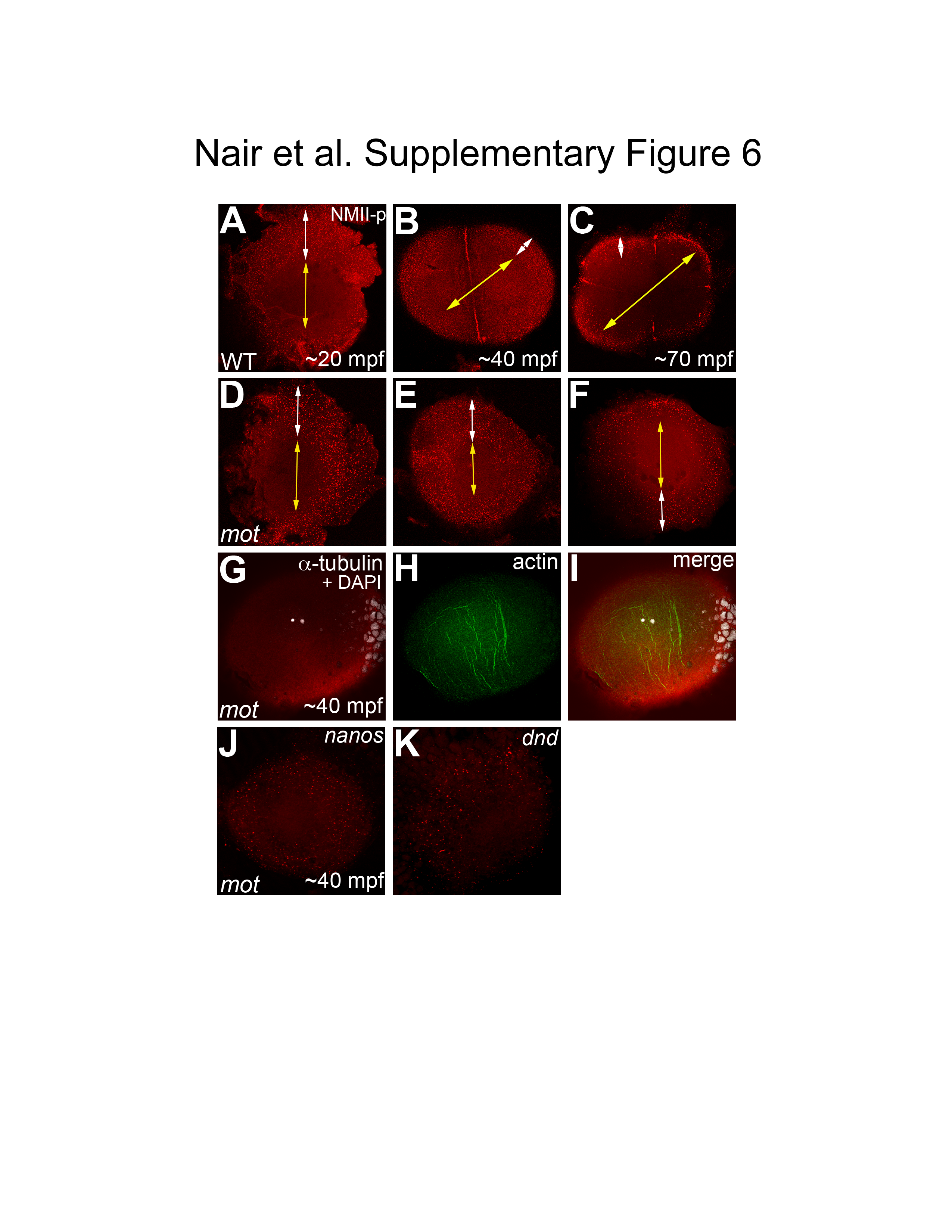

Supplement: Figure S6 — The cortical band of germ plasm RNPs fails to undergo a peripherally-directed compression in motley/birc5b mutants. Animal views of blastodisc cortex, immunolabeled with anti-NMII-p antibody. (A–F) Segregation of GP RNPs during the first two cycles. In wild-type embryos at 20mpf, the center of the blastodisc cortex is GP RNP-free as the RNPs are located in a broad peripheral band (A). As development proceeds, the blastodisc center remains free of GP RNPs and the peripheral band becomes compressed from the center outwards as the central GP RNP-free zone expands (B, C). In motley/birc5b mutants at 20mpf, the center of the blastodisc is GP RNP free and the RNPs are in a broad peripheral band as in wild-type embryos (D). However, the peripheral GP RNP band fails to further compress noticeably during development (E, F). Yellow double-headed arrows represent GP RNP-free zone in the blastodisc center, which does not expand in motley mutants. White double-headed arrows represent peripheral compression of GP RNP band in wild-type embryos, which does not occur effectively in motley/birc5b. (G–I) In motley mutants at a stage coincident with observed GP RNP segregation defects, F-actin (H,I) forms large bundles randomly crisscrossing the blastodisc, instead of circumferential bundles as observed in wild-type (Figure 5B, 5C, 5H; [16]). Such randomly oriented bundles also form in embryos after inhibition of microtubule polymerization [16] and may be related to F-actin gelation observed in early embryonic extracts [55]. DAPI labeling in (G,I) shows center of blastodisc; astral microtubules in G are not clearly visible as this time point coincides with the cyclical disassembly of this structure after reaching the cortex [16], [56]. (J,K) In situ hybridizations to detect germ plasm RNAs nanos (J) and dead end (K) in motley mutant embryos, showing defects in germ plasm segregation similar to those observed when visualizing GP RNPs with anti-NMII-p antibodies (compare to wild-type in [file pgen.1003448.s006.tif]
